# Isolation and culture of functional adult human neurons from neurosurgical brain specimens

**DOI:** 10.1093/braincomms/fcaa171

**Published:** 2020-10-17

**Authors:** Thomas I-H Park, Patrick Schweder, Kevin Lee, Birger V Dieriks, Yewon Jung, Leon Smyth, Justin Rustenhoven, Edward Mee, Peter Heppner, Clinton Turner, Maurice A Curtis, Richard L M Faull, Johanna M Montgomery, Michael Dragunow

**Affiliations:** Department of Pharmacology, Faculty of Medical and Health Sciences, The University of Auckland, Auckland, New Zealand; Centre for Brain Research, Faculty of Medical and Health Sciences, The University of Auckland, Auckland, New Zealand; Centre for Brain Research, Faculty of Medical and Health Sciences, The University of Auckland, Auckland, New Zealand; Department of Neurosurgery, Auckland City Hospital, Auckland, New Zealand; Centre for Brain Research, Faculty of Medical and Health Sciences, The University of Auckland, Auckland, New Zealand; Department of Physiology, Faculty of Medical and Health Sciences, The University of Auckland, Auckland, New Zealand; Centre for Brain Research, Faculty of Medical and Health Sciences, The University of Auckland, Auckland, New Zealand; Department of Anatomy and Medical Imaging, Faculty of Medical and Health Sciences, The University of Auckland, Auckland, New Zealand; Department of Pharmacology, Faculty of Medical and Health Sciences, The University of Auckland, Auckland, New Zealand; Centre for Brain Research, Faculty of Medical and Health Sciences, The University of Auckland, Auckland, New Zealand; Department of Physiology, Faculty of Medical and Health Sciences, The University of Auckland, Auckland, New Zealand; Department of Pharmacology, Faculty of Medical and Health Sciences, The University of Auckland, Auckland, New Zealand; Centre for Brain Research, Faculty of Medical and Health Sciences, The University of Auckland, Auckland, New Zealand; Department of Pharmacology, Faculty of Medical and Health Sciences, The University of Auckland, Auckland, New Zealand; Centre for Brain Research, Faculty of Medical and Health Sciences, The University of Auckland, Auckland, New Zealand; Department of Neurosurgery, Auckland City Hospital, Auckland, New Zealand; Department of Neurosurgery, Auckland City Hospital, Auckland, New Zealand; Department of Anatomical Pathology, LabPlus, Auckland City Hospital, Auckland, New Zealand; Centre for Brain Research, Faculty of Medical and Health Sciences, The University of Auckland, Auckland, New Zealand; Department of Anatomy and Medical Imaging, Faculty of Medical and Health Sciences, The University of Auckland, Auckland, New Zealand; Centre for Brain Research, Faculty of Medical and Health Sciences, The University of Auckland, Auckland, New Zealand; Department of Anatomy and Medical Imaging, Faculty of Medical and Health Sciences, The University of Auckland, Auckland, New Zealand; Centre for Brain Research, Faculty of Medical and Health Sciences, The University of Auckland, Auckland, New Zealand; Department of Physiology, Faculty of Medical and Health Sciences, The University of Auckland, Auckland, New Zealand; Department of Pharmacology, Faculty of Medical and Health Sciences, The University of Auckland, Auckland, New Zealand; Centre for Brain Research, Faculty of Medical and Health Sciences, The University of Auckland, Auckland, New Zealand

**Keywords:** dissociated neurons, cortical slice cultures

## Abstract

The ability to characterize and study primary neurons isolated directly from the adult human brain would greatly advance neuroscience research. However, significant challenges such as accessibility of human brain tissue and the lack of a robust neuronal cell culture protocol have hampered its progress. Here, we describe a simple and reproducible method for the isolation and culture of functional adult human neurons from neurosurgical brain specimens*. In vitro*, adult human neurons form a dense network and express a plethora of mature neuronal and synaptic markers. Most importantly, for the first time, we demonstrate the re-establishment of mature neurophysiological properties *in vitro*, such as repetitive fast-spiking action potentials, and spontaneous and evoked synaptic activity. Together, our dissociated and slice culture systems enable studies of adult human neurophysiology and gene expression under normal and pathological conditions and provide a high-throughput platform for drug testing on brain cells directly isolated from the adult human brain.

## Teaser

Neurosurgical specimens generated cultures of functional adult human neurons, astrocytes and microglia. Cultured neurons formed dense networks and demonstrated mature neurophysiological properties. These neuronal cultures provide the first easily generated dissociated functional human neurons and have many potential applications including in drug testing and neuroscience research.

## Graphical Abstract

**Graphical Abstract:**
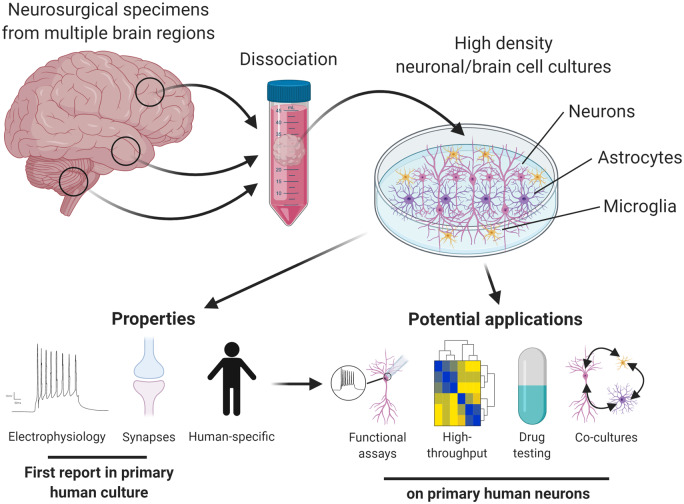

## Introduction

The ability to interrogate primary adult human neurons will greatly advance our understanding of the human brain (Silani *et al.*, 1988; Dragunow, 2008; Rice, 2012; Breyer *et al.*, 2015; Spaethling *et al.*, 2017), especially with evidence to suggest that many phenotypic and functional differences exist between the human brain and other animal model systems (Gibbons *et al.*, 2011; Seok *et al.*, 2013; Smith and Dragunow, 2014; Schwartz *et al.*, 2015; Napoli and Obeid, 2016; Spaethling *et al.*, 2017; Beaulieu-Laroche *et al.*, 2018). Human neocortical acute and organotypic brain slice cultures have been studied since the 1950s (Li and Mc, 1957), providing an *in vitro* model that can recapitulate regions of the human brain cytoarchitecture and functional circuits, but these do suffer from low throughput and difficulty in isolating individual cellular populations (Molnar *et al.*, 2008; Komlosi *et al.*, 2012; Verhoog *et al.*, 2013; Eugène *et al.*, 2014; Varga *et al.*, 2015; Jones *et al.*, 2016; Schwarz *et al.*, 2017, 2019; Beaulieu-Laroche *et al.*, 2018). The development of human-induced pluripotent stem cells provided an accessible human model system, but this technology is in its infancy and lacks an actual *in vitro* adult human neuronal model to benchmark and standardize their differentiation. To address these issues, attempts have been made to isolate and culture dissociated primary human neurons *in vitro* from neurosurgical specimens (Kim *et al.*, 1979; Brewer *et al.*, 2001; Brewer and LeRoux, 2007; Dragunow, 2008; Rice, 2012; Zhang *et al.*, 2016; Spaethling *et al.*, 2017), but low yields and the complexity of the isolation procedure have limited their use in neuroscience research. Only recently, researchers were able to conduct single-cell transcriptome analysis on primary human brain cells, which allowed for the delineation of not only different brain cell populations but also several neuronal subtypes (Darmanis *et al.*, 2015; Zhang *et al.*, 2016; Spaethling *et al.*, 2017). However, neurophysiological properties of these cultures were not reported, yet this is an essential readout of neuronal function. Here, we describe a standardized and simple primary adult human neuronal isolation procedure that results in viable, high-density neuronal cultures that express cortical neuronal markers. Also, we describe for the first time, the mature electrophysiological properties of functional primary adult human brain neurons and the presence of spontaneous currents and evoked synaptic connections between these neurons, some of which resemble those found in acute brain slice cultures. These findings make our culture system one of the most representative *in vitro* dissociated primary adult human neuronal model to date, and an ideal high-throughput platform to assess the effects of compounds on the function of primary adult human neurons.

## Materials and methods

### Collection of neurosurgical specimens

Fifty-one surgically excised brain tissues were obtained from patients undergoing neurosurgery (Supplementary Tables 1 and 6). Informed consent (by the Health and Disabilities Ethics Committee New Zealand) was obtained from all donors, and the brain tissue was transported from the operating room to the laboratory in ice-cold transport medium, Hibernate^TM^-A medium (Gibco), supplemented with 2% B27^®^ supplement (Invitrogen), and 10 µM ROCK inhibitor, Y-27632 2HCl (Selleckchem) in sterile falcon tubes (Falcon).

### Culture of primary adult human neurons

The grey matter regions from the cerebral cortex and the cerebellum were mechanically dissociated into <1 mm^2^ pieces prior to enzymatic digestion with the transport medium supplemented with 2.5 U/ml papain (Worthington) and 100 U/ml DNase I (Invitrogen) for 20 min at 37°C with gentle rotation. The digestion was halted by the addition of an equivolume of transport medium and any undigested tissue pieces were dissociated through gentle trituration. The cell suspension was passed through a 100-µm cell strainer, centrifuged (170 *g* × 7 min) and resuspended in neuronal growth medium (Dulbecco's Modified Eagles Medium (DMEM): F12, Gibco) supplemented with 2% B27, penicillin/streptomycin (Gibco), GlutaMAX^®^ (Invitrogen), 10 µM Y-27632 2HCl, 2 µg heparin (Sigma), 40 ng/ml of NGF, BDNF, NT-3, GDNF, and IGF-1 (Peprotech). Cells were plated onto poly-d-lysine-coated cell culture surfaces and maintained at 37°C with 5% CO_2_ and 99% humidity. Fifty per cent of the culture media was exchanged every 24 h for the first 48 h to remove cellular debris and toxic cellular by-products, then every 3–4 days, thereafter.

### Human cortex slice cultures

Cortical tissue (∼15–20 mm × 15–20 mm in size) was placed in ice-cold (4–8°C) *N*-methyl-d-glucamine protective cutting solution with the following composition (in mM): 93 *N*-methyl-d-glucamine, 2.5 KCl, 1.25 NaH_2_PO_4_, 30 NaHCO_3_, 20 HEPES, 25 glucose, 2 thiourea, 5 l-ascorbic acid, 3 Na-pyruvate, 0.5 CaCl_2_, 10 MgSO_4_⋅7H_2_O, (pH 7.4, osmolarity of 295–305 mOsm) (Fourie *et al.*, 2018; Ting *et al.*, 2018). Meningeal connective tissue, blood vessels or damaged regions were removed from the sample before slicing. Cortical tissue was sectioned at 250-μm thickness using a vibratome (Leica VT1200; Leica Biosystems, USA) in the same oxygenated and cooled cutting solution. The slices were transferred for recovery in a protective cutting solution at 34°C for 12–15 mins. Before culturing, 2–3 acute cortical slices were used for experiments as the days *in vitro* (DIV) 0 time point. The remaining cortical slices were transferred onto culture membranes (30 mm, 0.4-μm pores, Millipore) with 1 ml of culturing medium. The culturing medium was modified from published protocols (Eugène *et al.*, 2014; Le Duigou *et al.*, 2018), with the addition of 5 mM HEPES, and the replacement of glutamine with 0.5 mM GlutaMAX (Thermo Fisher). The medium was pre-equilibrated with gas (air/5% CO_2_) from the incubator before use. For equilibration, the culture medium was supplemented with HEPES (20 mM) for the first hour of culture in the incubator before changing the medium to one containing 5 mM HEPES. Slices were cultured at 37°C, 5% CO_2_ and 100% humidity, and the medium was changed daily.

### Immunohistochemistry

Biopsy specimens were fixed in 15% formalin and paraffin-embedded for immunohistochemistry examination. Seven-micrometre formalin and paraffin-embedded sections underwent antigen retrieval in a pressure cooker in Tris–Ethylenediaminetetraacetic acid (EDTA) buffer pH 9.0. The sections were permeabilized in phosphate-buffered saline (PBS) containing 0.1% triton X-100 (PBS-T) at 4°C and blocked in 10% normal goat serum in PBS for 1 h at room temperature (RT). Subsequently, the sections were incubated with primary antibodies (Supplementary Table 2) overnight at 4°C and then in corresponding goat AlexaFluor-conjugated secondary antibody (Invitrogen) at 1:400 for 3 h at RT. Finally, sections were incubated with Hoechst 33342 (Invitrogen) at 1:20 000 for 5 min at RT and then cover-slipped with ProLong gold antifade reagent (Invitrogen). All washes were done with PBS (3× 5 min). Control sections had primary antibodies omitted and showed no immunoreactivity (not shown). All confocal imaging was performed using an FV1000 confocal microscope (Olympus). Fluorescent recordings were performed with a MetaSystems VSlide slide scanner.

### Immunocytochemistry

Cells were fixed in 4% paraformaldehyde for 15 min at RT and permeabilized by 3× 10 min washes in PBS-T. Antibodies were dissolved in PBS-T with thiomersal and 1% normal goat serum at their designated dilutions (Supplementary Table 2) and incubated with the cells overnight at 4°C. Species-specific AlexaFluor-conjugated secondary antibodies were incubated for 3 h at RT. 5-Ethynyl-2′-deoxyuridine assay (Invitrogen) was conducted based on manufacturer’s instructions. Finally, the cells were incubated with a nuclear counterstain Hoechst 33258 (Sigma) for 30 min at RT and visualized using Metasystems ImageXpress fluorescence microscope (Molecular Devices) and the EVOS FL auto cell imaging systems (Thermo Fisher).

### Reverse-transcription polymerase chain reaction

Total RNA was extracted from the brain cell cultures (21–30 DIV) using an RNA isolation kit (RNAqueous^®^-Micro kit, Ambion) following manufacturer’s instructions. The extraction of RNA from brain tissues was conducted using Trizol^®^ (Ambion)-based extraction and an RNA extraction kit (RNeasy Mini Kit, Qaigen)-based purification methods. Complementary DNA was produced using Super-Script III First-Strand Synthesis kit according to the manufacturer’s instruction (Life Technologies, USA). In total, 5–20 ng of complementary DNA was used for each polymerase chain reaction (PCR) reaction using Taq DNA polymerase (Life Technologies, USA) for amplification of complementary DNA with primers sets listed in Supplementary Table 3. The PCR products were run on a 1.0% agarose gel and visualized with SYBR^®^ Safe DNA gel stain (Thermo Fisher) using the ChemiDoc gel imaging system (BioRad).

### Quantitative reverse-transcription polymerase chain reaction

Target gene expression levels were evaluated by quantitative reverse-transcription polymerase chain reaction (qRT-PCR) using a 7900HT Fast Real Time PCR system (Applied Biosystems, Singapore). The complementary DNA produced above was analysed for qRT-PCR using Platinum SYBR Green qPCR SuperMix-UDG with Rox kit (Invitrogen). The primers are detailed in Supplementary Table 4 and the relative changes were analysed according to the 2^−ΔΔCT^ method (Livak and Schmittgen, 2001). Each PCR run included a negative RT and non-template control, as well as melting curve assays to confirm specific product amplification. The plotted data represent the mean values of at least three independent experiments ± standard error of the mean (SEM).

### Nanostring^®^ nCounter analysis

Total RNA was isolated from both cortical tissue samples and the corresponding cultures at designated time points and processed for Nanostring^®^ nCounter analysis as per manufacturer’s instructions. Briefly, custom probes for the PlexSets were designed by Nanostring to incorporate 18 genes of interest and three housekeeping genes (Supplementary Table 5). The probes were ordered from Integrated DNA Technologies and run on a nCounter^®^ PlexSet^TM^ platform and analysed using the nSolver^TM^ Analysis software (Nanostring).

### Electrophysiology

For electrophysiological characterization, whole-cell patch-clamp recording techniques were conducted using a chamber-mounted Olympus BX51WI microscope under infrared differential interference contrast optics. Neurons were perfused with artificial cerebral spinal fluid containing (in mM): 119 NaCl, 2.5 KCl, 1.3 MgSO_4_, 2.5 CaCl_2_, 1 Na_2_HPO_4_, 26.2 NaHCO_3_, 11 glucose, equilibrated with 95% O_2_, 5% CO_2_ at room temperature. Standard borosilicate glass pipettes were pulled by a vertical glass capillary micropipette puller (PC-10, Narishige) to achieve a resistance of 5–7 MΩ when containing an internal solution comprising of (in mM): 126 K-gluconate, 4 KCl, 4 ATP-Mg, 0.3 GTP-Na_2_, 10 HEPES and 10 creatine phosphate at pH 7.25 at 300 total mOsm. A subset of cells were also filled with internal solution containing Alexa Fluor hydrazide 568 (1:500) and both differential interference contrast and fluorescence images were obtained for *post hoc* cell-type identification, characterization and morphological analysis. All signals were acquired using pClamp software (Molecular Devices) and analysed using Clampfit (Molecular Devices). Series resistance was monitored throughout the recordings, and those with series resistance changes of more than 20% and series resistance values higher than 15 MΩ were discarded from the analysis. The resting membrane potential (RMP) was measured in current-clamp configuration. The input resistance, membrane capacitance and time constant of the cells were calculated from recordings under voltage-clamp configuration where the cells were held at –70 mV and a protocol consisting of 20 ms hyperpolarizing −10-mV voltage steps was carried out. The ability to fire action potential(s) (APs) was recorded under current-clamp mode by injecting 1-s current steps that modulated membrane potentials from hyperpolarizing −90 or −100 to + 10 mV. Na^+^ and K^+^ channel properties were determined in voltage clamp at −70 mV with a 1-s 10-mV voltage steps ranging from −70 to +50 mV. AP threshold was measured from the slope of the phase-plane plot and AP-amplitude was calculated as a difference between the threshold and the peak of AP. AP after-hyperpolarization (AHP) amplitude was measured as the difference between the threshold of AP and the peak of maximal hyperpolarization after AP. AP half-width was measured at the half of AP-amplitude.

### Statistical analysis

Unless specified otherwise, all the results were derived from at least three independent brain tissue specimens. Combined or representative data are displayed as mean ± SEM where applicable. Statistical analysis was carried out using a Student’s *t*-test for comparing two groups, while a one-way or two-way analysis of variance (ANOVA) followed by Tukey’s multiple comparison test was conducted to compare between multiple groups. For data sets that carried unequal variances and large differences in absolute values, they were logarithmically transformed, tested for its validity and analysed with a general linear mixed model using R version 4.01 (R Core Team, 2020) and package lme4 (Bates *et al.*, 2015). Statistical significance was set at *P *<* *0.05. For qRT-PCR data, fold changes of 2 or more were considered significant (Livak and Schmittgen, 2001).

### Data availability

May be obtained from the corresponding authors upon reasonable request.

## Results

### Isolation and characterization of primary adult human neuronal cultures from neurosurgical specimens

**Figure 1:**
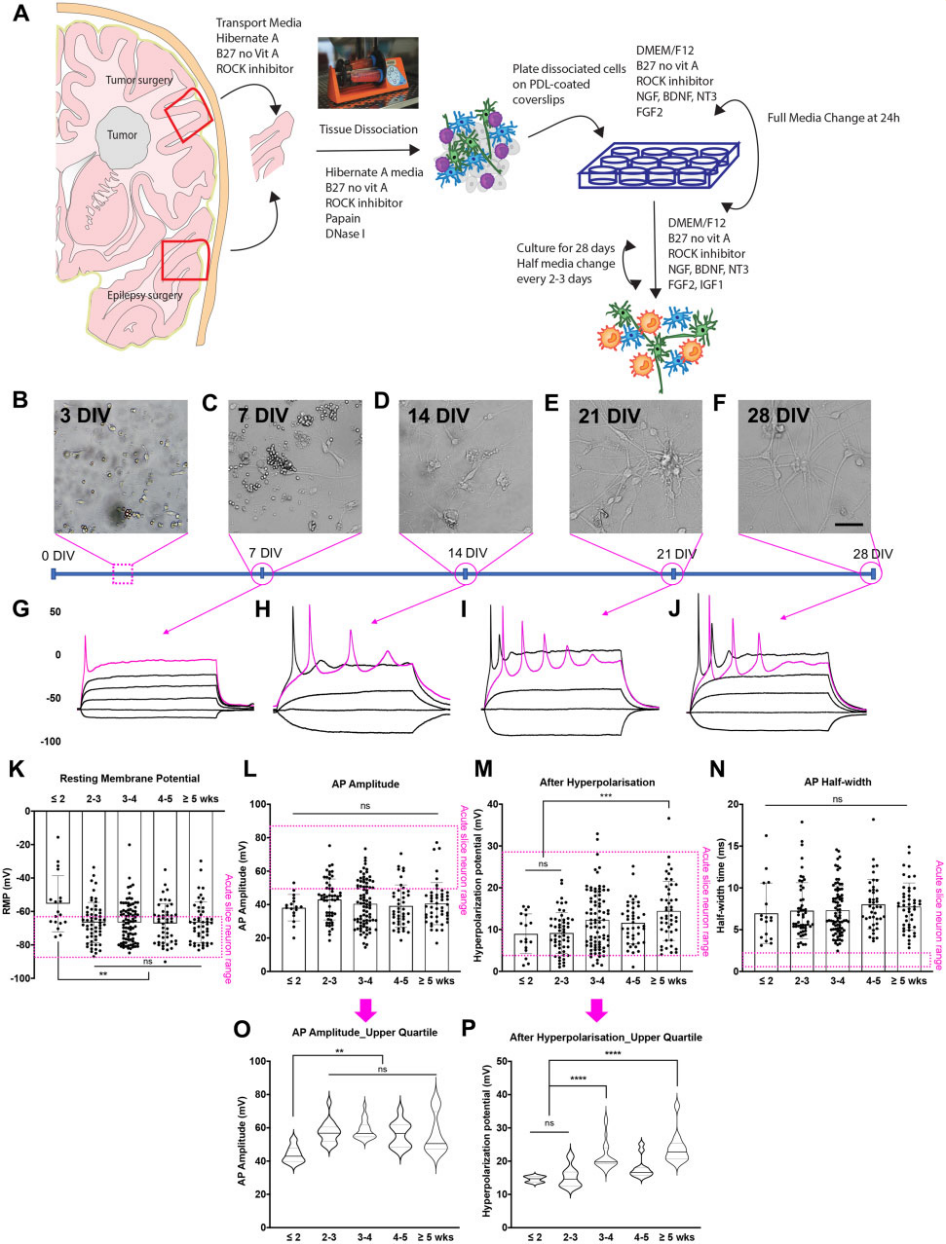
**Cells isolated from the adult human brain re-establish their neuronal phenotypes *in vitro.*** (**A**) Schematic for the isolation and culture process of adult human neurons from surgical specimens. (**B**–**F**) Live-cell images from a representative case at 2 (**B**), 7 (**C**), 14 (**D**), 21 (**E**) and 28 (**F**) DIV. (**G**–**J**) The corresponding electrophysiological traces and the analysis from cells patch clamped at 7 (**G**), 14 (**H**), 21(**I**) and 28 (**J**) DIV. (**K**–**P**) The RMP (**K**), AP-amplitude (**L**, **O**), AHP (**M**, **P**) and AP half-widths (**N**) were assessed at each timepoint from 14 DIV (2 weeks) onwards. The ≤5 weeks timeframe represents 35–70 DIV. The coloured box represents the value ranges recorded from the neurons in the adult human brain slice cultures. One-way ANOVA analysis with Tukey’s multiple comparison test was used. ns = *P *>* *0.05, **P <* *0.01, ***P <* *0.001 and ****P <* *0.0001. Scale bar = 20 µm

The adult human brain specimens were obtained from neurosurgical procedures that required the removal of non-neoplastic cortical tissue (Supplementary Fig. 1 and Table 1; Brewer *et al.*, 2001; Spaethling *et al.*, 2017). In order to determine the rate of maturation, a time-course experiment was conducted whereby the cultures were evaluated every 7 days for 28 days (Fig. 1B–J). The isolation process of dissociating cortical and cerebellar tissues into single cells resulted in the loss or damage of their neurite processes (Fig. 1B, 3 DIV). Over the next 28 DIV, neurons re-established their neurites (Fig. 1B–F) and developed mature electrophysiological properties (Fig. 1G–J and Table 1). From 14 DIV, active neurophysiological properties, such as repetitive-APs and synaptic activities, were recorded (Table 1). AP-amplitude and velocity (measured by AP half-width) were established by 14 DIV and were maintained throughout the rest of the culture period (up to 70 DIV). However, analysis of the upper quartile values of the AP-amplitudes at each culture period revealed that the number of neurons with larger AP-amplitudes continued to increase until 21 DIV and was then maintained throughout the rest of the culture (Fig. 1L and O). During the first 2 weeks *in vitro*, the RMP was more significantly depolarized [−55 ± 4 mV (*n* = 23) versus −67 ± 2 mV (*n* = 93), *P *<* *0.01], however, after 2 weeks *in vitro*, it maintained a more hyperpolarized level for the remainder of the culture period (over 5 weeks; Fig. 1K). The amplitude of the AHP significantly increased between 2 and 5 weeks *in vitro* (Fig. 1M and P). AP half-width, which provides a measure of AP velocity, was not significantly altered over the maturation period in culture (Fig. 1N). As cell morphology was the primary method for identifying neurons for whole-cell patch-clamp characterization, the apparent decrease in the percentage of AP-firing and synaptically active cells post 28 DIV could largely be attributed to a greater misidentification of astrocytes for neurons due to the development of more complex neurite-like processes in astrocytes (Table 1).

**Table 1:** Electrophysiological property changes of all the patch-clamp recorded cells at different culture durations

Electrophysiological properties	≤14 DIV (*n* = 26)	15–21 DIV (*n* = 93)	22–28 DIV (*n* = 165)	29–35 DIV (*n* = 127)	36–70 DIV (*n* = 145)
Resting membrane potential (mV)	−55 ± 4	−67 ± 2	−67 ± 1	−67 ± 2	−67 ± 2
Membrane resistance (MΩ)	357 ± 43	396 ± 60	271 ± 32	315 ± 43	304 ± 44
Cell capacitance (pF)	105 ± 18	190 ± 36	257 ± 41	151 ± 18	140 ± 18
% of AP-firing cells	69% (18/26)	61% (57/93)	52% (86/165)	37% (47/127)	33% (48/145)
% of AP-firing cells with repetitive-AP	11% (2/18)	21% (12/57)	26% (22/86)	19% (9/47)	6% (3/48)
% of AP-firing cells with synaptic activity	33% (6/18)	5% (3/57)	6% (5/86)	9% (4/47)	2% (1/48)

Both bright-field and immunocytochemistry (ICC) studies revealed that neurites progressively became more complex with increase in culture duration. When the expression of the neuronal marker MAP2 and the astrocyte marker GFAP was quantified, we found that the percentage of both MAP2^+^ and GFAP^+^ cells increased overall during the culture period (Supplementary Fig. 3A–L and Table 2). The percentage of MAP2^+^ cells increased to 21 DIV and plateaued, while the number of GFAP^+^ cells plateaued after 14 DIV. Furthermore, the percentage of GFAP^+^ cells were consistently lower than the MAP2^+^ cells in our cultures (Table 2). Gene expression data corroborated these findings, with both *MAP2* and *GFAP* transcripts increasing over time in our cultures relative to their 7 DIV expression levels (Supplementary Fig. 3M and N). The time-course quantification was done on six independent cases, while Supplementary Fig. 3 shows a representative case. Interestingly, 17 ± 3% of the total number of MAP2 and GFAP positive cells were double-positive (Supplementary Fig. 3I–L).

**Table 2:** Combined quantification data of MAP2^+^ and GFAP^+^ cells in our neuronal cultures

Culture duration	Percentage of MAP2^+^ cells and range (*n* = 6)	Percentage of GFAP^+^ cells and range (*n* = 6)
7 DIV	% = 9.82 ± 2.33 Range = 4.14–18.66%	% = 4.62 ± 2.60 Range = 0.46–16.00%
14 DIV	% = 13.88 ± 1.74 Range = 8.03–21.10%	% = 8.28 ± 3.85 Range = 2.37–27.14%
21 DIV	% = 22.92 ± 3.39 Range = 10.41–30.67%	% = 10.22 ± 3.02 Range = 4.05–24.76%
28 DIV	% = 20.21 ± 1.44 Range = 14.43–24.63%	% = 10.75 ± 3.48 Range = 2.73–26.21%

The average percentage and range of MAP2^+^ and GFAP^+^ cells in our cultures from six independent cases at 7-day intervals during the 28-day culture period. The percentage-positive values are an average of the six cases and shown as mean ± SEM. The range shows the lowest and the highest case for percentage-positive at each time point.

Although previous publications have demonstrated the validity of using peri-tumoural entry cortex for neuronal cultures (Brewer *et al.*, 2001; Spaethling *et al.*, 2017), we conducted further analyses to confirm the absence of tumour cell contamination in our specimens (Supplementary Figs 1 and 2). Immunohistological studies using the cell proliferation marker ki67 and stem cell/tumour cell markers, Nestin and Pax6, showed that these were present in the tumour, but not in the accompanying cortical region (Supplementary Fig. 1B–H). In addition, Nanostring^®^ analysis revealed that the peritumoral cortical specimens used in our culture had a high level of expression of neuronal and astrocytic genes, but lacked stem cell and progenitor genes (*PAX6*, *PROM1*, *NOTCH1*; *n* = 4; Supplementary Fig. 1I and J). Finally, it was evident when tumour cell contamination did occur, as the fast proliferating tumour cells rapidly occupied the cultures (Supplementary Fig. 2). These cultures were excluded from the study.

### Primary brain cell cultures express key pan-neuronal and neuronal sub-type-specific markers

**Figure 2:**
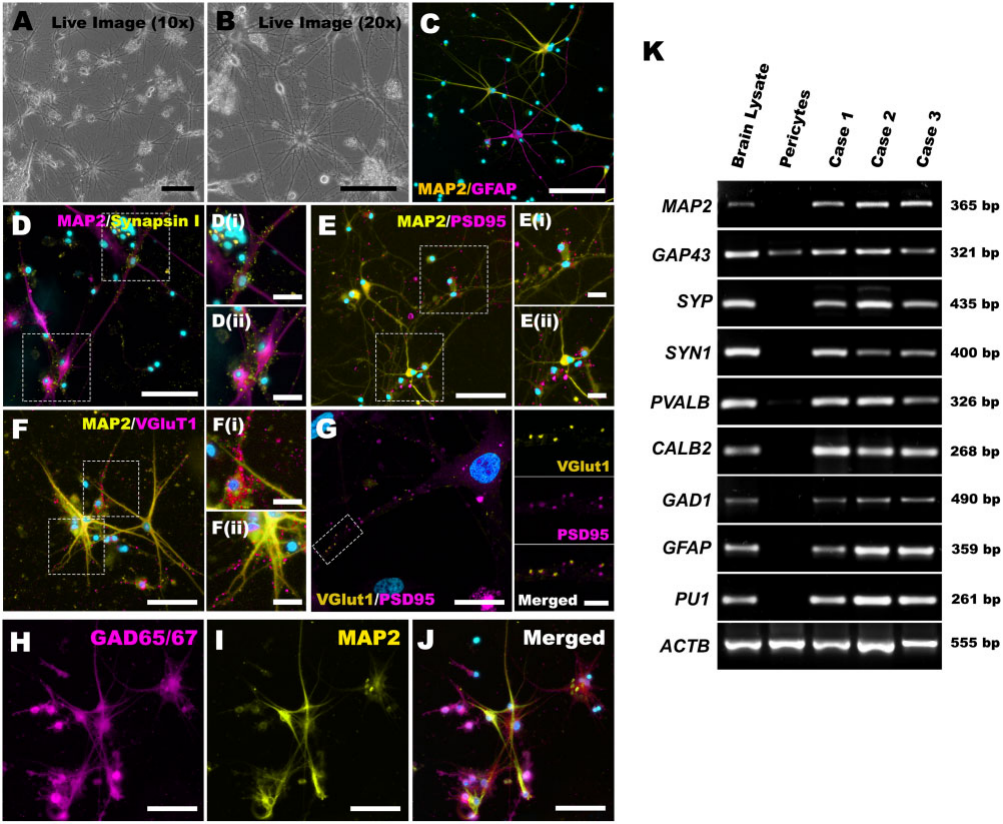
**The primary adult human neuronal cultures express mature neuronal and synaptic markers.** (**A**, **B**) Phase-contrast images from a live neuronal culture at 21 DIV. (**C**) Co-existence of neurons (MAP2^+^) and astrocytes (GFAP^+^) in the cultures. The MAP2^+^ neurons also expressed synaptic markers, synapsin I (**D**), PSD95 (**E**, **G**) and VGluT1 (**F**, **G**). They also labelled for the inhibitory neuronal marker, GAD65/67 (**H**–**J**). ICC studies were conducted on 19/46 cases and the representative cases are shown above. Three representative neuronal cultures from three different cases were processed for RT-PCR to probe for the presence of specific genes. The blots showed the amplification of most of the neuronal marker genes and some glial marker genes (**K**). These were also amplified in a representative brain lysate (positive control). The majority of the neuronal genes did not amplify in non-neuronal pericyte cultures (negative control). For the RT-PCR studies, Case 1 was superior frontal gyrus from an intra-ventricular meningioma surgery, Case 2 was a right parietal lobe resection from a GBM surgery and Case 3 was a temporal lobe resection as part of a temporal lobectomy surgery for epilepsy. The positive control was a biopsy temporal pole specimen obtained during a GBM surgery, and the negative control was a passage 7 pericyte culture derived from a temporal lobectomy surgery, similar to that of Case 3. Scale: **A**–**F** and **H**–**J** = 100 µm (insets = 20 µm); **G** = 20 µm (insets = 5 µm)

By 21 DIV, the majority of cells had extended elaborate neurite processes (Fig. 2A–C and Supplementary Fig. 4). The majority of the neurite-bearing cells expressed MAP2, while a subset of them expressed GFAP (Fig. 2C). The neurons also expressed excitatory pre- and post-synaptic markers, including synapsin I, VGluT1 and PSD95, evident as punctate co-localized synaptic labelling (Fig. 2D–G). Furthermore, the neurons expressed cell-type-specific markers, such as calretinin and GAD65/67 (Fig. 2H–J and Supplementary Fig. 4). Immunohistochemistry analysis of the corresponding cortical slice tissue also showed the expression of the same pan-neuronal and sub-type-specific markers present in our cultures (Supplementary Fig. 5). However, in the dissociated cultures, the percentage of neurons with individual cell-type-specific markers did vary between cases. For example, when the percentage of GAD65/67 expressing MAP2-positive neurons were quantified over three cases, one middle temporal gyrus culture showed 38.5% co-localization, while a cerebellar cortical culture resulted in 80.2% co-localization (mean = 52.8 ± 13.8%; *n* = 3).

The presence of different neuronal populations was further investigated from cultures maintained for 21–30 DIV using RT-PCR for cell-type-specific marker genes. As expected, our cultures contained cells that expressed pan-neuronal genes, *MAP2*, *GAP43*, synaptophysin (*SYP*) and synapsin I (*SYN1*; Fig. 2K). This corroborated our ICC studies. Furthermore, the cultures contained cells that expressed several interneuron sub-type-specific genes, such as parvalbumin (*PVALB*) and calretinin (*CALB2*). The presence of these inhibitory interneurons was verified by the amplification of a gamma-aminobutyric acid (GABA) synthesizing gene, *GAD1*. As shown in the ICC studies, all cultures showed amplification of an astrocyte marker gene, *GFAP*. The presence of CD45^+^ (Supplementary Fig. 4K and L) cells and *PU1* transcript amplification in all our cultures also indicated the presence of microglia in our cultures (Fig. 2K). A total of 10 separate primary brain cultures were processed for RT-PCR, and three representative cases from different surgical procedures and regions are shown in Fig. 2K. A positive control RNA directly isolated from a brain biopsy tissue and negative control of RNA extracted from a pure primary brain pericyte culture were also included (Park *et al.*, 2016).

### The cultured adult human neurons re-establish mature neurophysiological properties and synaptic connections

**Figure 3:**
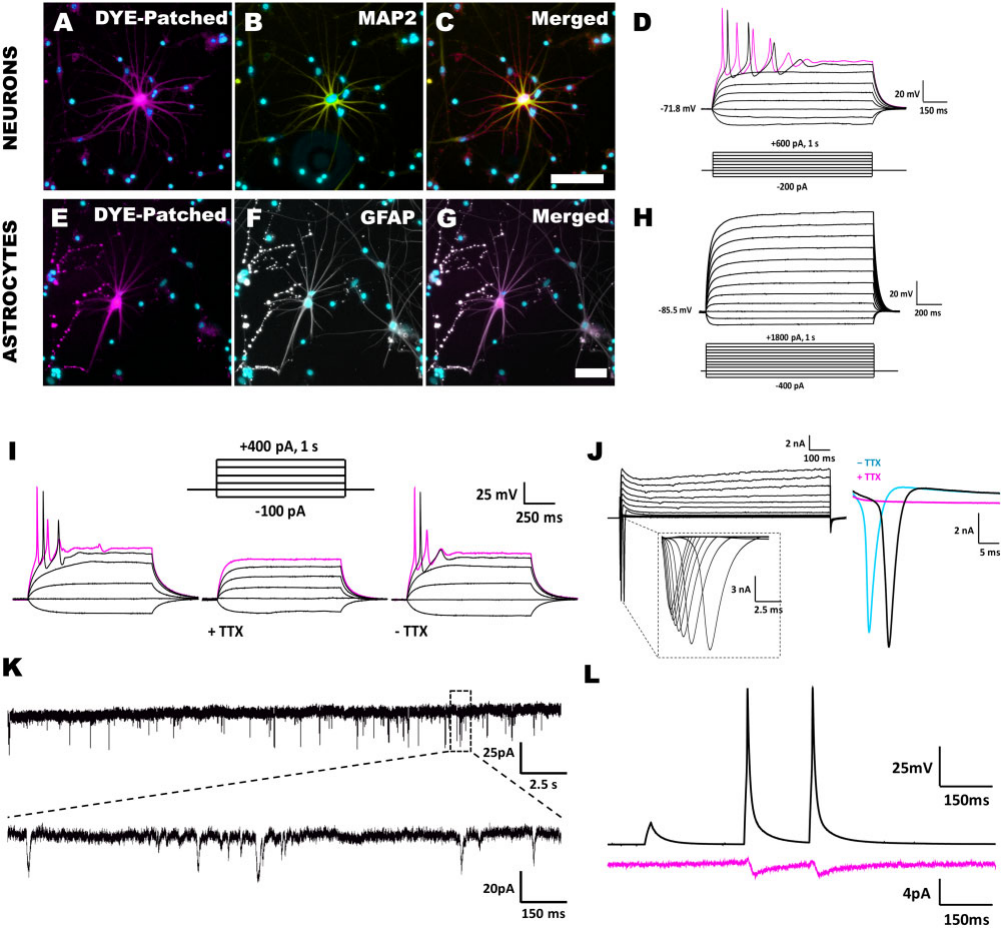
**Photomicrographs and whole-cell patch-clamp recordings demonstrate the presence of electrophysiologically active neurons in our primary adult human brain cell cultures.** A representative neuron injected with Alexa 568 dye during whole-cell patch-clamp recording (**A**) for post-recording analysis. This cell was found to express MAP2 (**B**, **C**) and fired repetitive-APs under current clamp with 1-s current pulses increasing in 100-pA increments from −200 pA (**D**; 23 DIV). A subset of cells did not fire APs in current-clamp recordings (**H**; 23 DIV). When dye-filled and analysed after whole-cell recording, this cell did not express MAP2 but did express GFAP (**E**–**H**). (**I**–**L**) Representative electrophysiological traces from whole-cell patch-clamped recordings (**I**; 28 DIV). Mature repetitive-APs that were blocked by the addition of tetrodotoxin (TTX; 1 µM). The APs reappeared upon TTX washout with artificial cerebral spinal fluid for 15 min (**J**; 28 DIV). A representative voltage-clamp trace showing the rapid and large inward current upon depolarization, which is again abolished in the presence of TTX and returned when TTX is washed out with artificial cerebral spinal fluid for 15 min. Voltage steps ranged from +10 to +120 mV in 10-mV increments (**K**; 14 DIV). Representative segment of spontaneous post-synaptic currents recorded in voltage clamp at −70 mV (**L**; 31 DIV). Representative traces from a paired whole-cell recording where post-synaptic currents were evoked in direct response to presynaptic APs, providing direct evidence of functional synaptic connections between adult human neurons *in vitro*. Scale: **A**–**C** and **E**–**G** = 100 µm

**Figure 4:**
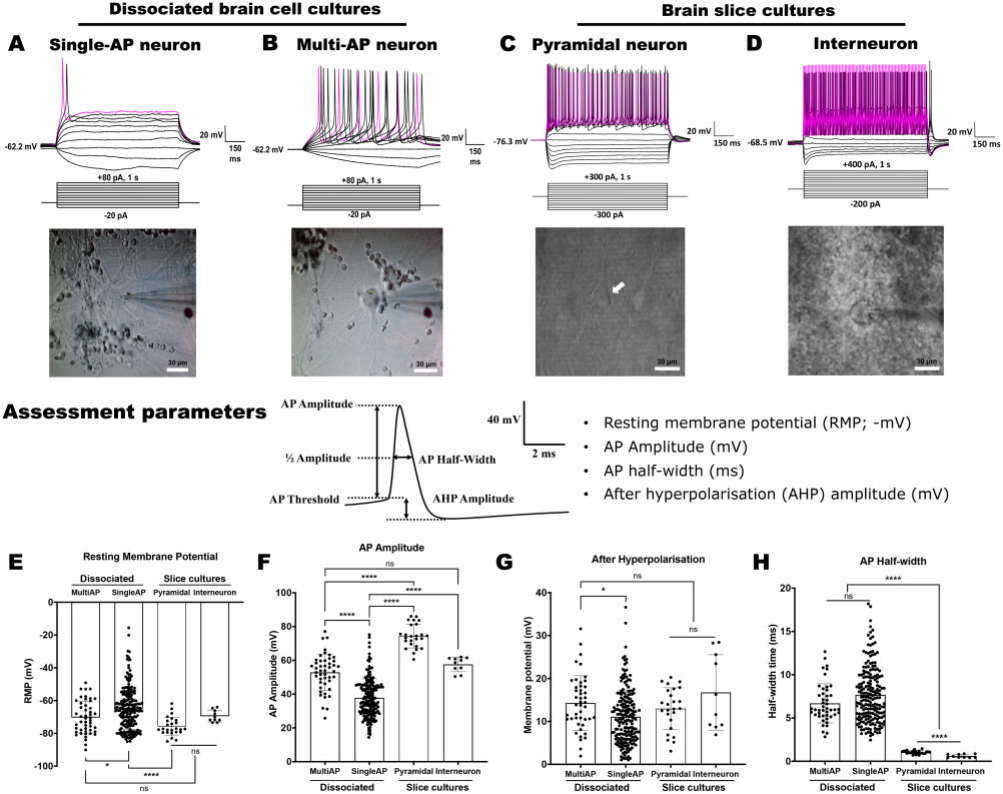
**A comparison of the electrophysiological properties of dissociated primary human neurons with human brain slice cultures.** (**A**–**D**) Representative whole-cell patch-clamp recordings and differential interference contrast images of a single- (**A**; DIV 31) and a repetitive-AP-firing (**B**; 23 DIV) dissociated neuron, and a pyramidal (**C**) and an interneuron (**D**) from slice cultures under current clamp in response to 1-s current steps. The white arrow in the differential interference contrast image of C indicates the layer 2/3 pyramidal neuron that was patch-clamp recorded. Scale: 30 µm. (**E**–**H**) The recorded neurons were categorized by AP-firing patterns and assessed for RMP (**E**), AP-amplitude (**F**), AHP amplitude (**G**) and AP half-width (**H**), as shown in the ‘Assessment parameters’ illustration. The dissociated neurons that elicited repetitive multi-firing APs and those that only elicited single-AP were compared to pyramidal-like and interneuron-like neurons recorded from cortical slice cultures. For data with equal variance, a one-way ANOVA analysis with Tukey’s multiple comparison test was employed, and where variance was different, logarithmically transformed data were analysed using a general linear mixed model. ns = *P *>* *0.05, *P <* *0.05 and ****P <* *0.0001

Functional neurons display active membrane properties and form synaptic connections. However, to date, no electrophysiological data on dissociated primary adult human neurons *in vitro* exist. To address this, we conducted whole-cell patch-clamp recordings from more than 600 viable cells; of these, 449 cells had stable membrane properties that were fit for electrophysiological analysis (summarized in Table 3A). The isolated brain cells exhibited active neurophysiological properties by 15 DIV and remained functional until 70 DIV (Supplementary Table 7). Of the 449 cells recorded, 260 cells were classified as neurons based on their ability to fire at least a single rapid AP (Table 3B), with example traces shown in Figs 3 and 4. Furthermore, 48 of the 260 AP-firing cells generated repetitive-APs (Table 3B and Fig. 3A–D). When the basal membrane properties of the neurons were compared to those of AP-silent cells (Table 3A), the AP-firing neurons were more depolarized (−65.5 ± 0.8 mV versus −75.3 ± 0.6 mV, *P *<* *0.001), exhibited greater input resistance (316.0 ± 20.7 MΩ versus 206.7 ± 31.7 MΩ, *P *<* *0.01) and had a greater cell capacitance value (192.1 ± 16.8 pF versus 145.5 ± 21.1 pF, *P *<* *0.05). Furthermore, both the basal and active membrane properties of the neurons isolated from tumour-associated cases and those from non-tumour cases (e.g. epilepsy) were indistinguishable (Supplementary Tables 8 and 9). During whole-cell patch-clamp recordings, a subset of cells were filled with AlexaFluor hydrazide dye in order to identify the cells post-recording [AP-firing (*n* = 20) and AP-silent (*n* = 15)]. The cells that fired APs were found to be MAP2^+^, further confirming their neuronal status (Fig. 3A–D). On the other hand, the dye-filled AP-silent cells were co-labelled with the astrocytic marker, GFAP (Fig. 3E–H). More specifically, all neurons elicited single or repetitive-APs upon the injection of depolarizing currents (Fig. 3D) and displayed large fast-activating inward currents evoked by a series of voltage steps (Fig. 3J). When neurons were grouped into single-AP and repetitive-AP-firing cells, the latter showed a more hyperpolarized RMP, lower input resistance and increased cell capacitance (Table 3B). Irrespective of the neuronal type, AP-firing was blocked by bath application of tetrodotoxin (1 µM) (Fig. 3I and J), indicative of sodium channel-mediated APs, and these recovered after a 15-min washout with artificial cerebral spinal fluid. These neurons also formed functional synapses as seen by spontaneous inward post-synaptic currents at −70 mV (Fig. 3K). Furthermore, evoked post-synaptic currents could be recorded in the paired whole-cell configuration when the presynaptic neuron evoked APs, showing functional synaptic transmission between neurons. Evoked synaptic currents were observed in 27% of the cells that showed spontaneous post-synaptic currents (Fig. 3L). Together, our electrophysiological and imaging data demonstrate that functionally active primary adult human neurons can be isolated from neurosurgical specimens and cultured *in vitro*.

**Table 3:** Electrophysiological properties recorded from primary human brain cells with whole-cell patch clamp

(A) Basal membrane properties of all the patch-clamped cells			
	Action potential firing cells (*n* = 260)	Action potential silent cells (*n* = 189)	Significance (*P*-value)
Resting membrane potential (mV)	−65.5 ± 0.8	−75.3 ± 0.6	<0.001 (***)
Input resistance (MΩ)	316.0 ± 20.7	206.7 ± 31.7	<0.01 (**)
Cell capacitance (pF)	192.1 ± 16.8	145.5 ± 21.1	<0.05 (*)

(B) The basal membrane properties of only tde AP-firing cells			

	Repetitive-AP (*n* = 48)	Single-AP (*n* = 212)	Significance (*P*-value)

Resting membrane potential (mV)	−70.4 ± 1.4	−64.4 ± 1.2	<0.05 (*)
Input resistance (MΩ)	214.6 ± 38.3	339.0 ± 31.2	<0.05 (*)
Cell capacitance (pF)	337.0 ± 65.5	159.2 ± 17.8	<0.001 (***)

(A) The membrane properties were categorized into AP-firing cells and AP-silent cells. The resting membrane potentials of AP-silent cells were significantly more hyperpolarized and exhibited a lower input resistance. Cell capacitance measurements were similar for both subtypes of cells. (B) The membrane properties of AP-firing cells when classified into repetitive-AP-firing and single-AP-firing neurons. When classified by AP-firing status, the repetitive-AP-firing cells exhibited significantly more hyperpolarized resting membrane potentials, lower input resistance and a higher cell capacitance when compared to the single-AP-firing cells.

### Repetitive multi-AP-firing neurons exhibited the most mature electrophysiological properties

Acute and organotypic slice cultures have long been the gold-standard for assessing *in vitro* electrophysiological properties of brain cells. To further investigate the extent of the electrophysiological maturity achieved by our dissociated neuronal cultures, we compared their basal and active electrophysiological properties to pyramidal- and inter-neurons recorded from human brain slice cultures (Fig. 4). Pyramidal neurons were identified morphologically by their distinct inverted triangular pyramidal cell body shape with long extended dendritic structures under differential interference contrast microscopy (Fig. 4C). Fast-spiking interneurons were identified electrophysiologically based on their intrinsic physiological properties, such as high frequency firing of short APs (half-width of fast-spiking interneuron = 0.6073 ± 0.06278 ms, *n* = 10; AP half-width of pyramidal neurons = 1.038 ± 0.03788, *n* = 25; *P*-value < 0.0001; general linear mixed model) without adaptation in firing rate, and large amplitude after hyperpolarization that was comparable to previous studies (Fig. 4D–H) (Wang *et al.*, 2016; Schwarz *et al.*, 2019).

The dissociated neurons were compared to the slice cultured neurons for the following parameters: (i) RMP, (ii) AP-amplitude, (iii) AHP and (iv) AP half-width. Multiple categorical groups were compared to the pyramidal and interneurons from the human cortical slice cultures, including single versus multi-AP-firing neurons (Fig. 4), paediatric versus adult culture-derived neurons and neocortex versus cerebellar cortex-derived neurons (Supplementary Fig. 6). We observed that there were no significant differences in any the above parameters between the paediatric and adult-derived dissociated neurons (Supplementary Fig. 6A–D). In addition, no differences were observed in the percentage of cells capable of repetitive firing of multiple APs and synaptic activity (Supplementary Table 10). When compared together to neurons from human cortical slice cultures, however, dissociated neurons exhibited more immature properties, with significantly more depolarized RMP, smaller AP-amplitudes and longer AP half-widths (Fig. 4E–H).

We also compared neuronal properties in cultures prepared from different brain regions. A comparison of neocortex- and cerebellar cortex-derived dissociated neurons revealed that the cerebellar neurons exhibited significantly higher AP-amplitudes and shorter AP half-widths, both of which are suggestive of a more mature neurophysiological state. Despite these differences, both the neocortical and cerebellar neurons, as a collective, remained significantly less mature in the electrophysiological properties compared with that observed in the human cortical slice cultures (Supplementary Fig. 6E–H).

It is important to highlight the variability we observed in electrophysiological properties in the dissociated neuronal cultures (Fig. 4), likely evident of a range of neuronal subtypes and maturation stage. In order to determine whether the neurons exhibiting more mature electrophysiological properties were similar to mature neurons in cortical slices, we grouped neurons by their AP-firing status of repetitive multi-AP-firing or single-AP-firing cells. We observed that the multi-AP-firing neurons in dissociated cultures showed comparable AP-amplitude, RMP and AHP to those in interneurons from slice cultures and comparable RMP and AHP to pyramidal neurons in slice cultures. The AP half-widths of the neurons from the dissociated cultures, however, were significantly longer when compared to the neurons recorded from the slice cultures (Fig. 4E–H).

## Discussion

A reliable, well characterized and electrophysiologically active primary adult human neuronal culture system is instrumental for the understanding of human neurological diseases. In this study, we provide a highly reproducible and simple method that achieves the above. We have comprehensively characterized the cells using an array of neuronal markers, which largely represented those seen in neurons *in situ*. Furthermore, we conducted electrophysiological studies on over 400 isolated brain cells from multiple brain regions and showed that for the first time, primary adult human neurons can re-establish their neurophysiological properties *in vitro* (summarized in Supplementary Table 8). This was possible by first designing a simple and rapid protocol (<1 h) to increase neuronal viability. The addition of a Rho kinase inhibitor to the transport medium decreased neuronal apoptosis during the transport and the isolation process (Watanabe *et al.*, 2007), and later, aided with neurite outgrowth during the regrowth process (Sagawa *et al.*, 2007; Gu *et al.*, 2013; Leong *et al.*, 2015). We further modified this medium by supplementation with multiple neurotrophic factors (NGF, NT-3, BDNF and GDNF) and IGF-1 to aid in neuronal survival (Lindholm *et al.*, 1996; Culmsee *et al.*, 2002; Pascual *et al.*, 2008), stimulate neurite outgrowth (Dijkhuizen and Ghosh, 2005; Hollis *et al.*, 2009; Carlson *et al.*, 2014) and enhance synaptic connectivity (Shen and Crain, 1994; Kakizawa *et al.*, 2003; Carlson *et al.*, 2014). Using this protocol, neurons were cultured from all 51 specimens obtained from 49 patients, irrespective of region, age, underlying pathology or gender. We consistently achieved high-density neuronal cultures that expressed many cortical brain cell markers, including neuronal markers, MAP2, GAP43 and NeuN (Jacobson *et al.*, 1986; Mullen *et al.*, 1992; Dehmelt and Halpain, 2005), astrocytic marker, GFAP (Hol and Pekny, 2015) and microglial marker CD45 (Rustenhoven *et al.*, 2016). The MAP2-positive cells also co-localized with synaptic markers, synapsin I and VGluT1 (Wojcik *et al.*, 2004), indicating the presence of glutamatergic synapses. Inhibitory neuronal markers GAD65/67 (GAD2/1) (Bu *et al.*, 1992; Wojcik *et al.*, 2004), as well as several cortical interneuron sub-type-specific markers such as calretinin, calbindin and parvalbumin (Kim *et al.*, 2014) could also be observed. These data support that both excitatory glutamatergic and inhibitory GABAergic synapses are present in these cultures. These results also corroborate findings from single-cell transcriptome studies of isolated (Darmanis *et al.*, 2015) and cultured primary human brain cells (Spaethling *et al.*, 2017), which contained a population of cells with high expression of neuronal markers such as calbindin, GAD1 and parvalbumin.

Furthermore, primary cultures from specific brain regions were observed to result in the growth of different neuronal populations (e.g. calretinin-positive granule cells from the cerebellar tissue). Another interesting observation was the apparent selection bias of our culture procedure towards the inhibitory neuronal phenotypes. All the cultures we analysed through ICC and RT-PCR contained a large number of cells expressing inhibitory interneuron markers, but not always the excitatory markers. There are several explanations for this phenomenon. One possibility is that excitatory projection neurons (such as the pyramidal neurons) have long processes that can be severely damaged during the isolation process, while the smaller interneurons mainly project within the grey matter region and experience less axonal damage (Markram *et al.*, 2004). Another possibility is that our culture medium contains glutamine, which can break down to form glutamate in solution and cause excitotoxicity in more vulnerable neurons, such as the large excitatory projection neurons (Driscoll *et al.*, 1993; Mattson and Magnus, 2006). Indeed, such phenomenon has been reported in rodent primary neuronal cultures where components in the media selectively caused excitotoxicity in NMDA receptor expressing neurons (Driscoll *et al.*, 1993; Hogins *et al.*, 2011). Furthermore, even in human neurodegenerative diseases, there is a tendency for the larger projection neurons with low calcium binding proteins to be more vulnerable to oxidative stress and excitotoxicity (Mattson and Magnus, 2006; Wang and Michaelis, 2010).

It is inevitable that surgically obtained human brain tissues have underlying pathologies (Brewer *et al.*, 2001; Brewer and LeRoux, 2007; Darmanis *et al.*, 2015; Spaethling *et al.*, 2017). As a number of our cultures were isolated from peri-tumoural tissue, we verified that our neurons did not differentiate from tumour cells, as in the original peri-tumoural brain tissue a lack of tumour cell contamination was demonstrated through immunohistochemistry and gene expression studies. Also, no significant differences in electrophysiological properties were observed between the neurons isolated from peri-tumoural and non-neoplastic cortical specimens. Culture contamination with tumour cells, if present, was readily apparent as the tumour cells proliferated and soon dominated the post-mitotic neuronal culture (Blain, 2010), and these cases were not included in this study. It is therefore highly unlikely that the neurons reported in this study originated from tumour cells. The possibility of NPC differentiation also appears to be unlikely. Using immunohistochemistry and Nanostring^®^ analyses, we found insignificant levels of NPC marker genes in tissue. Furthermore, the stem cell marker gene, *CD133*, and NPC marker gene *PAX6*, which are regulated during the neurogenesis process (Coskun *et al.*, 2008; Kallur *et al.*, 2008), were found in extremely low levels in tissue and remained unchanged throughout our neuronal culture process *in vitro*. The 5-ethynyl-2′-deoxyuridine cell proliferation assay also found that the mitotic cells were almost all non-neuronal, which included brain pericytes (Park *et al.*, 2016), endothelial cells (Smyth *et al.*, 2018) and, to a lesser extent, microglia (Rustenhoven *et al.*, 2016). The very small percentage of 5-ethynyl-2′-deoxyuridine^+^MAP2^+^ cells may indicate a minor NPC population; however, the more likely scenario is, like the other thymidine analogues, 5-ethynyl-2′-deoxyuridine was incorporated into DNA during DNA repair and chromatin remodelling in mature neurons (Kuan *et al.*, 2004; Taupin, 2007; Zheng *et al.*, 2011). Further, there is limited evidence for the presence of NPCs in the adult human cortex (Au and Fishell, 2006; Huttner *et al.*, 2014), and we and others have demonstrated that differentiating primary adult human NPCs into high-density neuronal cultures were only possible by first expanding the NPCs from neurogenic regions, such as the subventricular zone (Kukekov *et al.*, 1999; Westerlund *et al.*, 2003; Park *et al.*, 2012).

To the best of our knowledge, this study provides the first account of electrophysiological recordings from dissociated primary adult human neurons *in vitro* (summarized in Table 4). Thus far, neurophysiological studies on adult human neurons have been limited to acute brain slice preparations or organotypic slice cultures (Molnar *et al.*, 2008; Komlosi *et al.*, 2012; Verhoog *et al.*, 2013; Eugène *et al.*, 2014; Varga *et al.*, 2015; Jones *et al.*, 2016; Schwarz *et al.*, 2017, 2019; Beaulieu-Laroche *et al.*, 2018). In this study, we found that by 21 DIV that neurons could re-establish many of their neurite processes and elicit highly depolarizing and rapid APs (Contreras, 2004). Importantly, the presence of spontaneous synaptic activity and evoked post-synaptic currents in response to presynaptic APs signify that our cultures enable the formation of synapses between isolated adult human neurons. Of the AP-firing neurons, those that elicited repetitive-APs exhibited a more hyperpolarized RMP, lower input resistances, exhibited APs with greater amplitudes relative to single-AP neurons, all of which indicates a more mature neuronal phenotype (Westerlund *et al.*, 2003). Acute and organotypic human brain slice cultures are recognized as the gold-standard *ex vivo* model for the human brain, as it largely maintains the cytoarchitecture and the functional connectivity seen *in vivo* (Molnar *et al.*, 2008; Komlosi *et al.*, 2012; Verhoog *et al.*, 2013; Eugène *et al.*, 2014; Varga *et al.*, 2015; Jones *et al.*, 2016; Schwarz *et al.*, 2017, 2019; Beaulieu-Laroche *et al.*, 2018). Hence, it was encouraging to see that the repetitive-AP-firing neurons re-established many of the electrophysiological properties seen in the slice cultures, albeit at relatively lower levels of maturity, especially with regards to AP velocity. Our current model, therefore, affords researchers to utilize dissociated functional human neuronal cultures for biomedical characterization and disease-modelling purposes that do not currently require full neurophysiological maturity. Further, this system provides a characterized platform for investigating and improving the neuronal maturation of both the adult and paediatric primary human neurons.

**Table 4:** Comparison of previously reported primary adult human neuronal cultures

Tissue source	Region	ICC characterization	Molecular characterization	Electrophysiology	References
Post-mortem	Trigeminal and superior cervical ganglia	Bright-field images and EM only	NA	Extracellular recordings	Kim *et al.* (1979)
Surgical biopsy brain tissue	Caudate	Bright-field images only	NA	NA	Silani *et al.* (1988)
Surgical biopsy brain tissue	Mainly frontal and temporal lobe	∼40% neurofilament^+^ cells	NA	NA	Brewer *et al.* (2001)
Surgical biopsy brain tissue	Various regions of the human neocortex	Tuj1 immunostaining only	Bulk population RNA-seq	NA	Zhang *et al.* (2016)
Surgical biopsy brain tissue	Various regions of the human neocortex	Bright-field images only	Single cells RNA-seq	NA	Spaethling *et al.* (2017)
Surgical biopsy brain tissue	Various regions of the human neocortex and the cerebellum	Up to 20% MAP2^+^ cells + synapsin I and many other neuronal sub-type-specific markers	Many neuronal genes detected by Nanostring^®^ and amplified by both RT-PCR and qRT-PCR	Paired whole-cell patch-clamp recordings Fired mature AP and formed active synapses	This study

To date, there are few reported electrophysiological properties of human paediatric brain-derived neurons *in vitro*—either from brain slices or from dissociated cultures*.* Here, we have successfully isolated neurons from both paediatric and adult tissue sources, and to our surprise, in our dissociated cultures, we found no significant differences in any of the electrophysiological parameters assessed between the isolated neuronal populations. Even between the slice cultures, the only significant difference was seen in the magnitude of the post-AP hyperpolarization, with neurons from the adult slices showing a greater level of hyperpolarization. Therefore, our data reveal significant difference between human and other rodent model systems in that by post-natal stages in human brain development, the non-synaptic neurophysiological properties appear established and continue to be maintained into adulthood.

The availability of neurosurgical specimens from multiple brain regions afforded the opportunity to investigate any differences between neurons isolated from the neocortex and the phylogenetically older cerebellar cortex. The cerebellar cultures generally resulted in higher neuronal density, which is logical given the fact that the cerebellum has roughly four times the number of neurons compared to the neocortex (Herculano-Houzel, 2010). Furthermore, cerebellar cultures had a significantly higher proportion of neurons that fired repetitive multi-APs (Supplementary Table 8), which from our results above, supports a more mature neuron. This again could be attributed to the higher neuronal density, as previous non-human neuronal culture studies have shown that higher neuronal densities resulted in greater neuronal survival and maturation (Banker and Cowan, 1977; Kaech and Banker, 2006). As our brain slice cultures were conducted using neocortical specimens, any direct comparison of cerebellar and cortical neuronal properties in the non-dissociated form could not be conducted. Although outside the scope of this manuscript, future studies aiming at systematically investigating the neurophysiological properties of neurons from different regions of the human brain would be of great interest to the neuroscience community.

The neurite-bearing cells that did not elicit APs were most likely astrocytes (Dallerac *et al.*, 2013; Chai *et al.*, 2017). Other than the added advantage of investigating primary human astrocytes *in vitro*, the presence of astrocytes in our cultures is likely to aid in neuronal survival, maturation and synapse formation (Dallerac *et al.*, 2013; Zhang *et al.*, 2016). Zhang *et al.* exquisitely isolated primary human astrocytes by utilizing a novel immunopanning protocol and demonstrated that co-culturing astrocytes with rodent retinal ganglion cells vastly improved the neuronal survival and formation of synapses (Zhang *et al.*, 2016). This study also isolated primary human neurons but only reported ICC and RNA-seq data; hence, it would be interesting to investigate, using their immunopanning methods, whether astrocytes do indeed influence the survival and synaptic maturation of primary human neurons. Interestingly, we also observed a number of cells were immunoreactive for both MAP2 and GFAP antibodies in our human dissociated cultures. Although MAP2 and GFAP recognize neuronal dendrites and astrocytes, respectively, and have been considered mutually exclusive *in vivo*, under *in vitro* conditions, they have been shown to be co-expressed by differentiating immature neurons (Laywell *et al.*, 2005; Park *et al.*, 2012) or reactive astrocytes (Geisert *et al.*, 1990). However, the traditional notion of MAP2 and GFAP mutual exclusivity has been challenged *in vivo* (Hol *et al.*, 2003). Importantly, this co-localization only occurred in a small subset of the MAP2^+^ cells with the majority of cells being either MAP2^+^ or GFAP^+^.

In summary, we have established a functional adult human neuronal culture system that can provide a valuable model system to examine the dynamic functional aspects of human cellular neurophysiology and gene expression *in vitro* in a high-throughput manner. This offers the exciting possibility of modelling human neurological diseases in genetically un-modified adult human neurons to find therapeutic avenues for patients with brain disorders.

## Supplementary material

Supplementary material is available at *Brain Communications* online.

## Supplementary Material

fcaa171_Supplementary_Data

## Abbreviations

AHP =: after-hyperpolarization
AP =: action potential
DIV =: days *in vitro*
ICC =: immunocytochemistry
qRT-PCR =: quantitative reverse-transcription polymerase chain reaction
RMP =: resting membrane potential
