# Supplementary material for: Isolation and culture of functional adult human neurons from neurosurgical brain specimens
Source: Brain Commun. 2020 Oct 17;2(2):fcaa171. doi: 10.1093/braincomms/fcaa171 (PMC7660143; doi:10.1093/braincomms/fcaa171)
Supplement: fcaa171_Supplementary_Data [file fcaa171_supplementary_data.pdf]

Supplementary Information

Supplementary Figure 1.

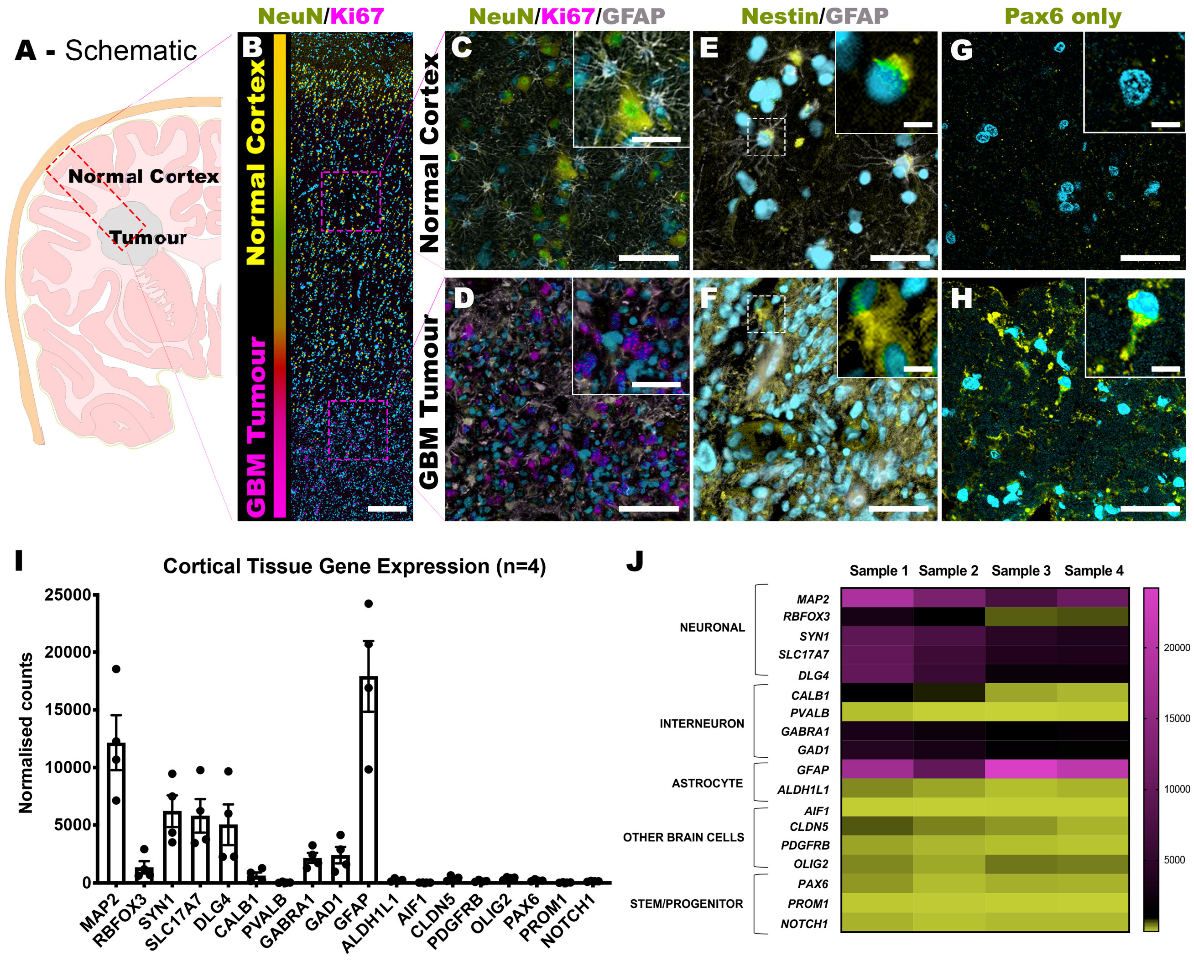

The isolation of neuronal cultures from non-neoplastic cortical tissues excised during tumour surgeries. A schematic illustrating the source of the resected cortical and tumour tissue (A). IHC analysis of the cortical tissue for the neuronal marker, NeuN (yellow) and the proliferating cell marker, ki67 (magenta; B). The outer cortical region was devoid of any proliferating ki67^{+} cells (B and C), while the tumour region had large numbers of ki67^{+} proliferating (B and D) cells. The cortical region also contained normal brain cells expressing neuronal marker NeuN (B and C), astrocyte marker GFAP (grey; C) and lacked progenitor marker Nestin (E) and Pax6 (G), while the tumour region contained significant numbers of ki67^{+}, GFAP^{+}, Nestin^{+}, Pax6^{+} tumour cells (D, F, and H). I-J: Nanostring nCounter® analysis of four surgically resected cortical tissues used for neuronal culture. The average gene counts for the four cases are shown as a bar graph (I), while the expression levels of individual cases are shown as a heat map (J). Scale: B = 200 μm, C-D = 100 μm, E-H = 50 μm, insets: C-D = 50 μm, E-H = 10 μm.

Supplementary Figure 2.

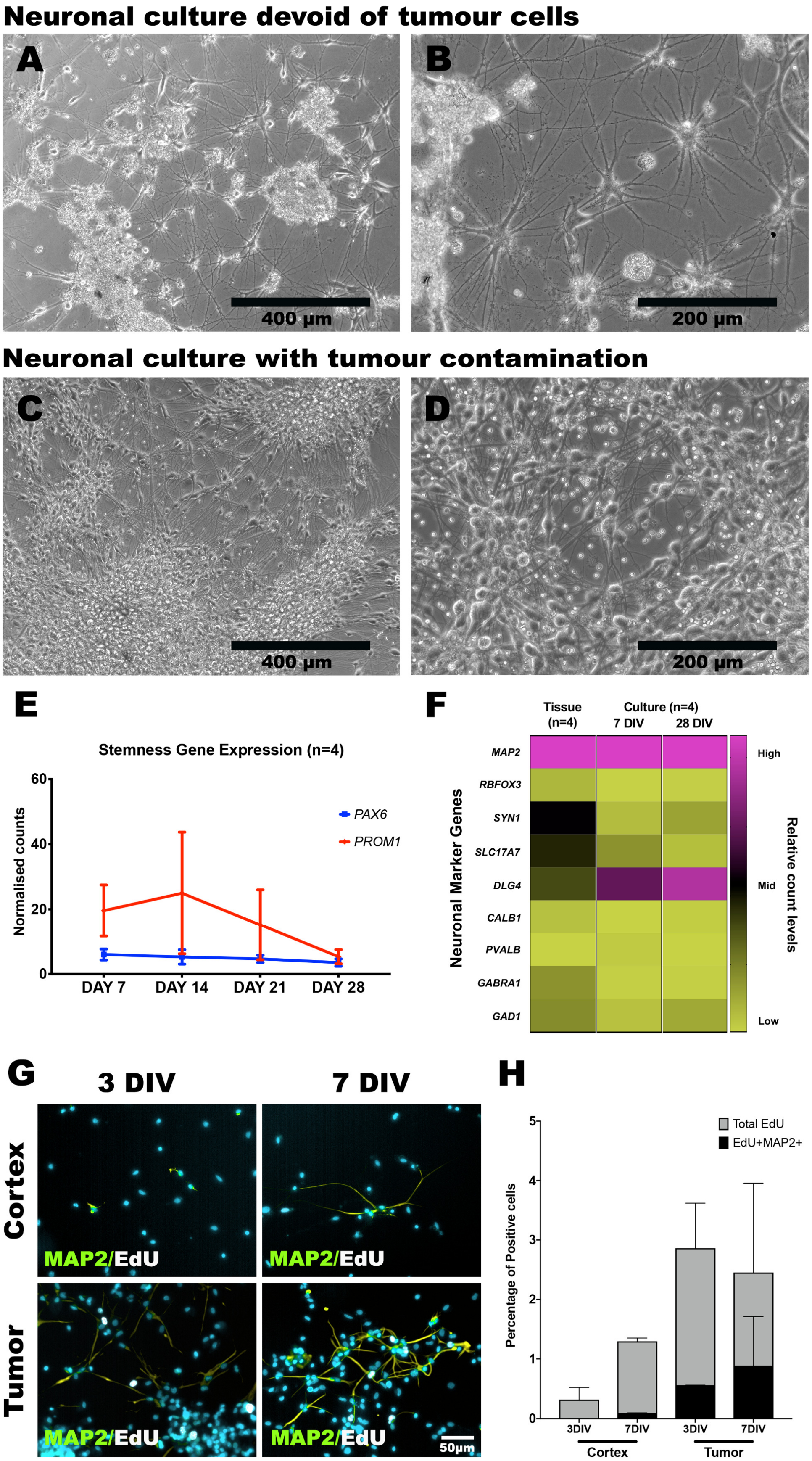

The neuronal cultures are devoid of any detectable tumour cell contamination. Photomicrographs of neuronal cultures showing a high density of neurite-possessing cells in the absence (A-B) or presence of tumour cell contamination (C-D). Gene expression analysis of cultures devoid of tumour cell contamination using Nanostring® also showed low counts of stem cell and neural progenitor cell marker genes that were not regulated throughout our culture period (E), and the pattern of neuronal marker gene expression was similar in cultured cells to those of tissue (F). Panels G-H shows the relatively low levels of EdU^+ proliferating cells in the cortical cultures compared to the patient-matched tumour cultures, as well as the near absence of EdU^+MAP2^+ cells in the cortical cultures.

Supplementary Figure 3.

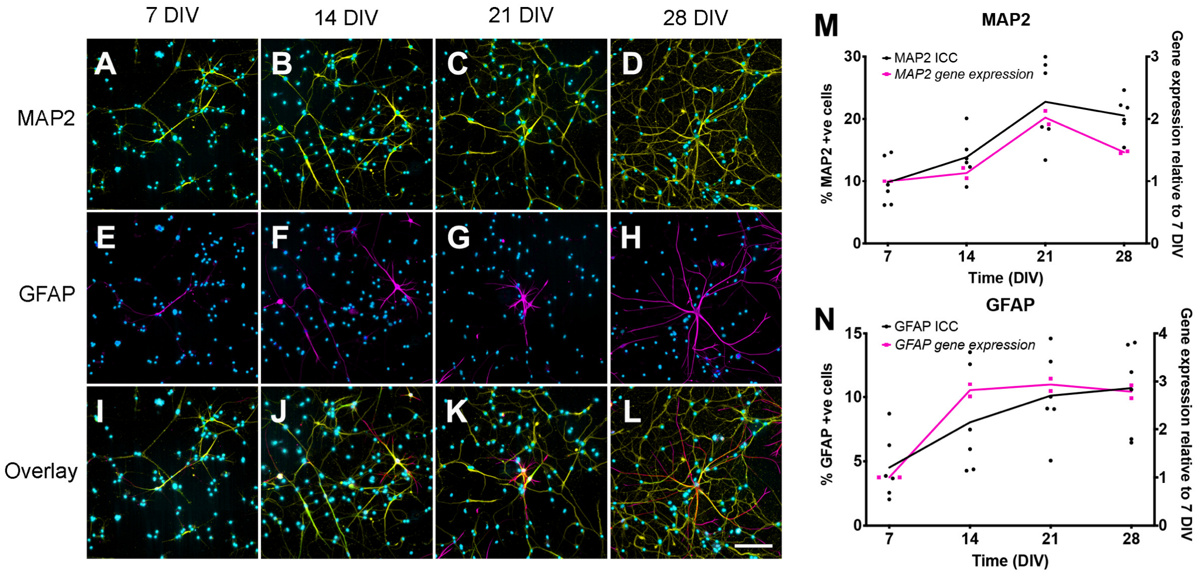

Cells isolated from the adult human brain re-establish their neuronal and astrocytic phenotypes *in vitro*. A-L: ICC images of MAP2 (A-D) and GFAP (E-H)-positive cells and their merged images (I-L) over the 28-day culture period. M-N: Image quantification of MAP2^{+} (M) and GFAP^{+} (N) cells in six cultures (n=6) and the gene expression changes of *MAP2* and *GFAP* in two of these cases (n=2). Scale bar:100 µm.

Supplementary Figure 4.

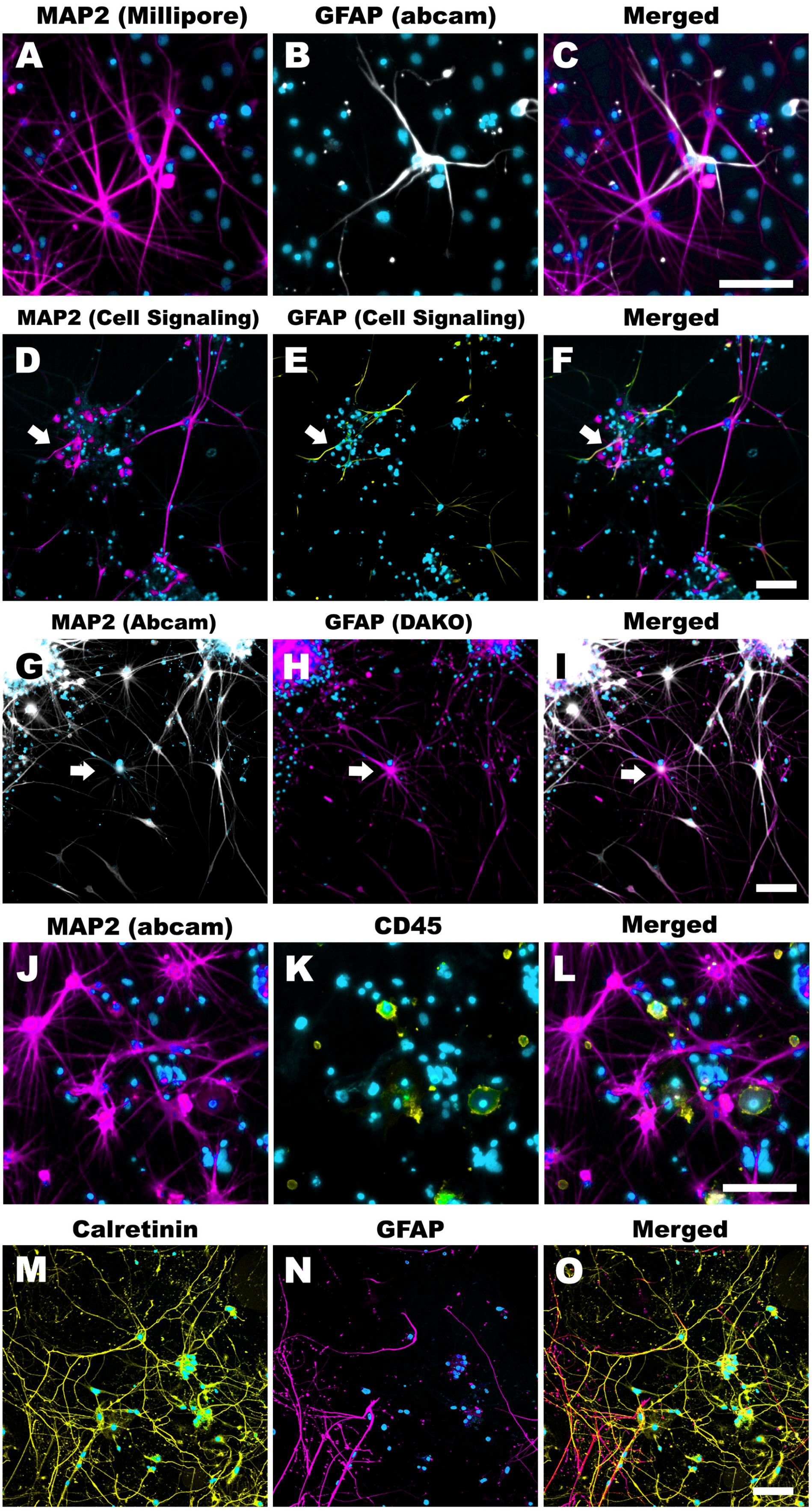

Cells isolated from the adult human brain contain neurons, astrocyte and microglia. Photomicrographs illustrating neuronal cultures ($MAP2^+$) that contain both $GFAP^+$ astrocytes and $CD45^+$ microglia. Several different combination MAP2 and GFAP antibodies were trialled and all showed MAP2 only and GFAP only cells, but also those co-expressing MAP2 and GFAP (white arrows). The cultures also contained $CD45^+$ microglia that did not co-localise with any of the neuronal markers tested. The interneuron marker, Calretinin, also labelled a large number of neurons. All images were taken from cultures at 28 DIV. Scale: 100 µm.

Supplementary Figure 5.

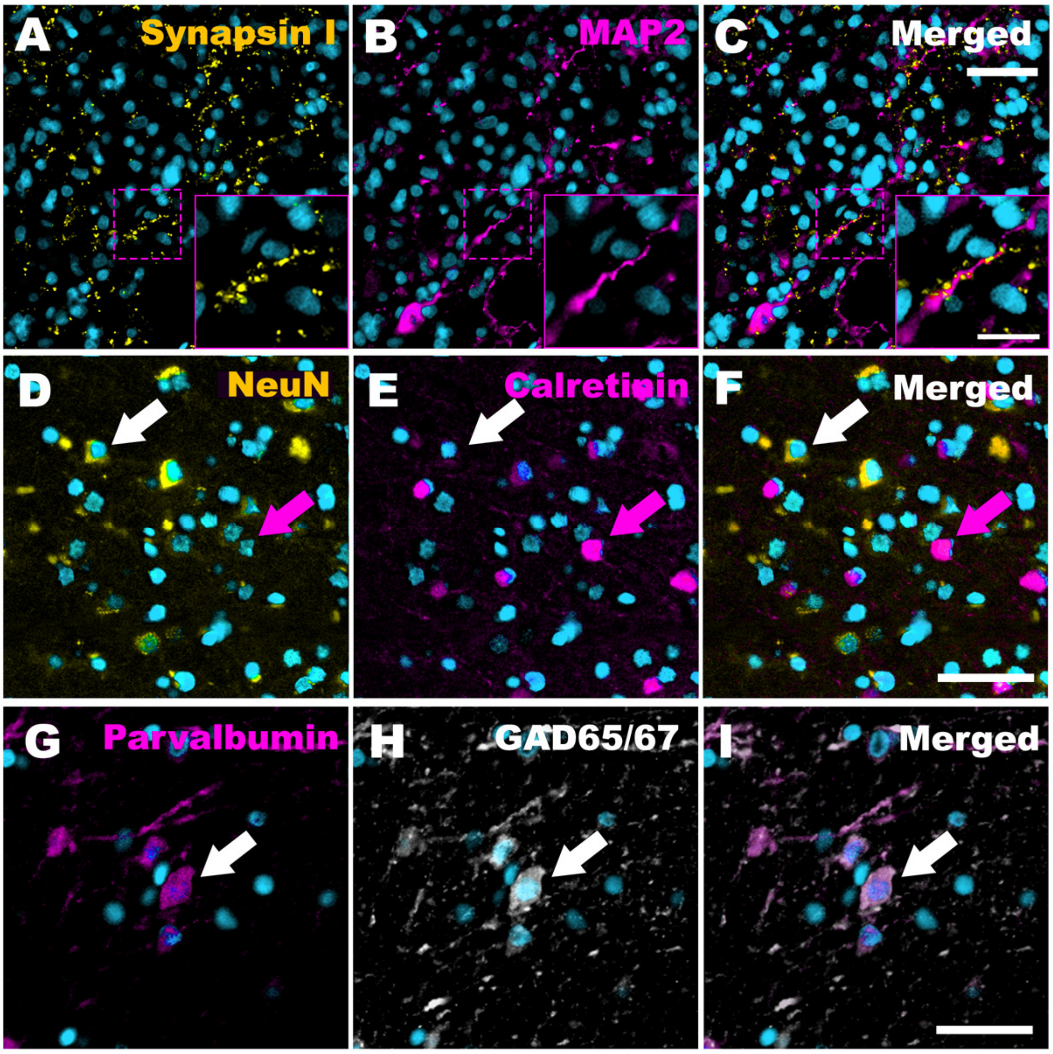

Cortical slice tissue contained the same neuronal markers expressed in the neuronal cultures. FFPE tissue from the same specimen as were cultured, were probed for the markers detected in neurons *in vitro*. The presynaptic marker, synapsin I, exhibited punctate staining onto MAP2^{+} processes (A-C). Another neuronal marker, NeuN and an interneuron marker calretinin were both present in the cortical specimens (D-F). Parvalbumin (G) and GAD65/67 (H) were also present and frequently co-localized onto a single cell (I). IHC studies of neuronal markers in the corresponding cortical tissue was conducted in 15 of the 37 specimens received and the representative case is shown in Figure S3. Scale bar = 100 µm, inset = 10 µm.

Supplementary Figure 6.

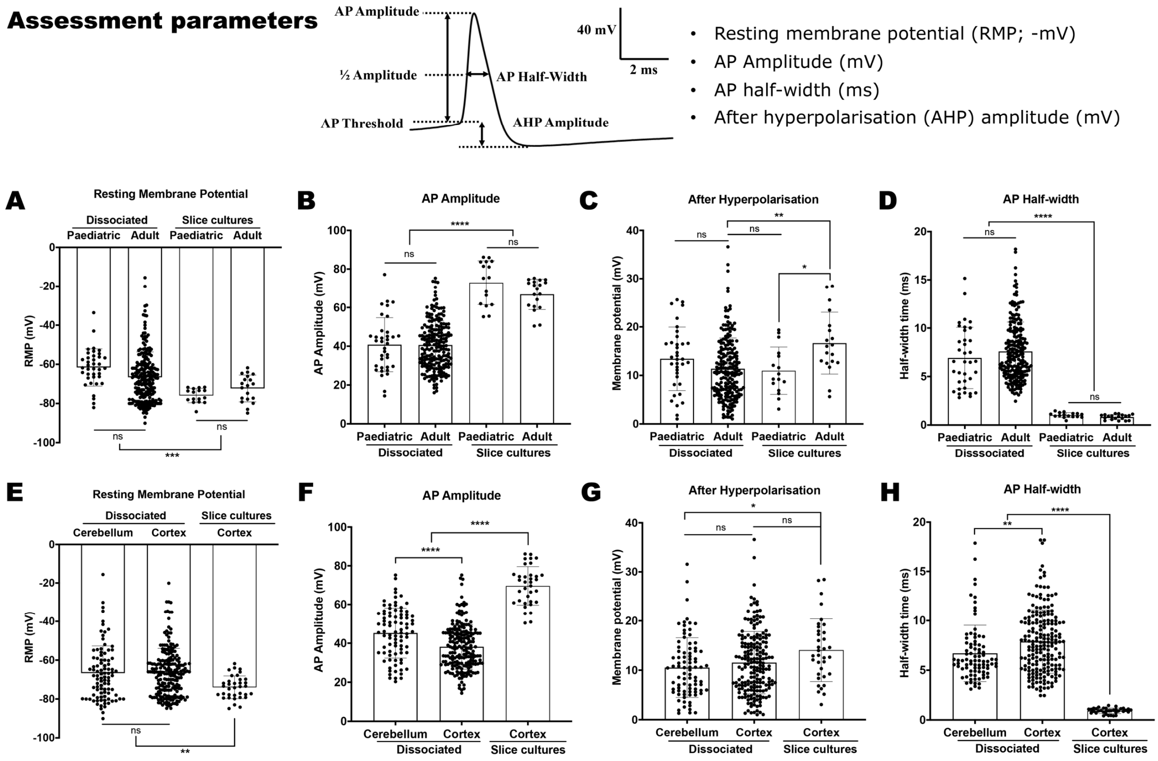

A comparison of the electrophysiological properties of dissociated primary human neurons with human brain slice cultures. The recorded dissociated primary neurons were categorised by age (A-D) and brain region (E-H), and assessed for RMP, AP amplitude, after-hyperpolarisation amplitude, and AP half-width, as shown in the **'Assessment Parameters'** illustration. These properties were also compared to equivalent neurons recorded from brain slice cultures. **Panels A-D** compare the neurons recorded from the dissociated cultures and the cortical slice cultures from paediatric and adult cases. **Panels E-H** compare the neurons cultured from dissociated cerebellar and neocortical tissues, as well as neurons recorded from neocortex-derived slice cultures. For data with equal variance, a One-way ANOVA analysis with Tukey's multiple comparison test was employed, and where variance were different, logarithmically transformed data was analysed using a general linear mixed model. ns = $p >$ 0.05, * = $p < 0.05$, ** = $p < 0.01$, *** = $p < 0.001$, and **** = $p < 0.0001$.

Supplementary Figure 7.

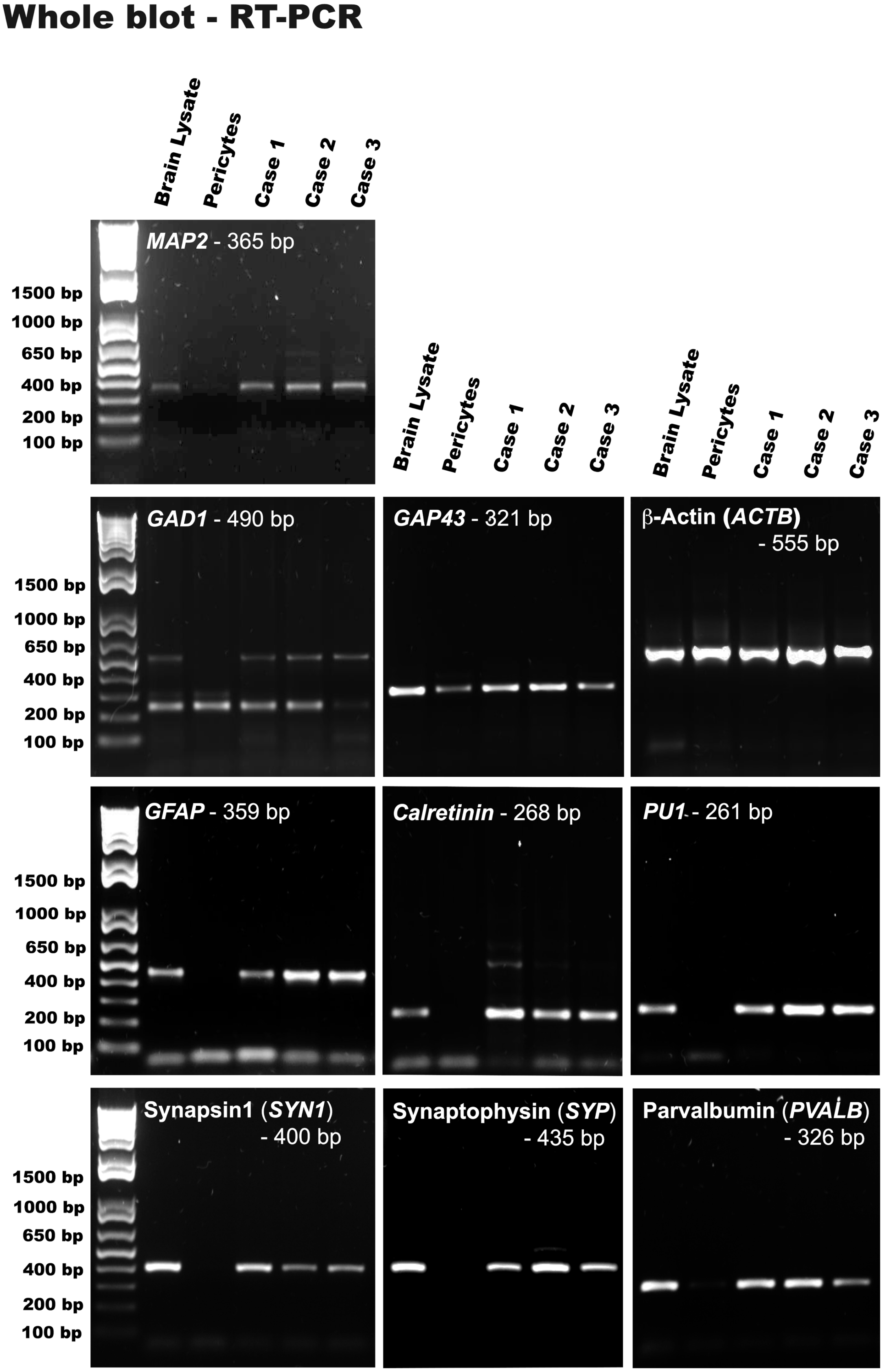

Full sized blots for the RT-PCR experiments conducted for Figure 2K on whole-brain, pericyte (negative control) and three representative neuronal cultures.

Supplementary Tables

Supplementary Table 1. Neurosurgical specimens used for the neuronal cultures. Different regions of the human cortex were obtained as part of the required neurosurgical procedure for the neurological disorders listed. 51 specimens were obtained from 49 patients with an average age of 42 ± 3.5 years.

Underlying pathology	Cortical region	Number of specimens
Primary brain tumours – high grade	Frontal lobe	6
(WHO grades III-IV)	Temporal lobe	5
GBM, anaplastic astrocytoma III	Occipital/Parietal lobe	5
Primary brain tumours – low grade	Frontal lobe	1
(WHO grades I-II)	Temporal lobe	2
	Cerebellum	3
Metastatic brain tumours	Frontal lobe	2
	Temporal lobe	1
	Cerebellum	4
Intractable epilepsy	Temporal lobe	10
	Hippocampus	2
Paediatric cases - cortical dysplasia and	Frontal lobe	5
cortical tubers	Parietal lobe	5
		n = 51

Supplementary Table 2. Antibodies used for immunolabelling

Antibody	Species	Company	Catalog #	ICC dilution	IHC dilutions
**MAP2**	Mouse	Millipore	MAB3418	1:500	1:500
**MAP2**	Rabbit	Millipore	Ab5622	1:500	
**MAP2**	Chicken	Abcam	Ab5392	1:500	
**NeuN**	Rabbit	Millipore	ABN78	1:500	1:250
**Calretinin**	Rabbit	Swant Laboratories	7696	1:2,000	1:2,000
**GAD65/67**	Rabbit	Abcam	Ab26116	1:1,000	1:1000
**Parvalbumin**	Guinea Pig	Synaptic Systems	195004	1:2,000	1:2000
**ki67**	Mouse	DAKO	M7420	-	1:250
**GFAP**	Chicken	Abcam	Ab4674	1:20,000	1:3,000
**GFAP**	Mouse	Cell Signaling	3670	1:10,000	
**GFAP**	Rabbit	DAKO	Z0334	1:10,000	
**Synapsin I**	Rabbit	Sigma Aldrich	S193-10UG	1:200	1:200
**vGlut-1**	Guinea Pig	Synaptic Systems	135304	1:200	-
**CD45**	Mouse	Abcam	Ab8216	1:500	
**Nestin**	Mouse	Millipore	MAB5326	1:500	1:500
**PSD95**	Mouse				

Supplementary Table 3. List and sequence of primers used for RT-PCR experiments

**Gene name**<br>**Accession number**	**Primer sequence**<br>**(Forward/reverse)**	**Start**<br>**BP**	**Stop**<br>**BP**	**Amplicon**<br>**Length**
ACTB	AGCACGGCATCGTCACCAACT	215	235	555 bp
**NM_001101**	AGCGGAACCGCTCATTGCCA	769	750	
MAP2	TCCAGTTTCTGCGCCCA	213	229	365 bp
**NM_001039538**	GACAAGTCCTTCCCCTGCTC	577	558	
GAP43	GCTGTGCTGTATGAGAAGAACC	389	410	321 bp
**NM_001130064**	GCGGGGTGGCATAATTCAGA	709	690	
SYP	GCCAACAAGACCGAGAGTGA	188	207	435 bp
**NM_003179**	GTTGAGTCCCGAGGTCACAG	622	603	
SYN1	TGAAGCCGGATTTTGTGCTG	662	681	400 bp
**NM_006950**	ACACGCACGTCATATTTGGC	1076	1057	
PVALB	CTCTGCCCGCTCAAACAGTT	6	25	326 bp
**NM_001315532**	CAACCCCAATTTTGCCGTCC	331	312	
CALB2	AGATGTCCCGACTCCTGCCT	604	623	268 bp
**NM_001740**	ACAATCTCCAGGTCCTTGCG	871	852	
GAD1	GCAAAACCGTGAGCTGGATT	1	20	490 bp
**NM_000817**	TTAGTGGTATTGGGGTCCGC	490	471	
GFAP	CAGTTATCAGGAGGCGCTGG	1032	1051	359 bp
**NM_001131019**	CAAAGCGCCGTGTCTGAGAG	1390	1371	
PU1	AATGTCAAGGGAGGGGGCTC	57	76	261 bp
**NM_001080547**	GGCGTTGGTATAGATCCGTGT	317	297	

Supplementary Table 4. List and sequence of primers used for qRT-PCR experiments

**Gene name**<br>**Accession number**	**Primer sequence**<br>**(Forward/reverse)**	**Start**<br>**BP**	**Stop**<br>**BP**	**Amplicon**<br>**Length**
ACTB	TGGTGGGCATGGGTCAGAAGGA	131	152	94 bp
**NM_001101**	ATGCCGTGCTCGATGGGGTACT	224	203	
MAP2	TCGCAGAGCAGGGAAGAGTGGT	849	870	80 bp
**NM_001039538**	AACTTGGTGGGGTGCCAGGAGT	928	907	
GFAP	TGACCGCTTTGCCAGCTACATCG	231	253	69 bp
**NM_002055**	TCAGCAGCCAGCGCCTTGTTT	299	279	

Supplementary Table 5. List and sequence of probes used for Nanostring® nCounter experiments

**Gene name**<br>**Accession number**	**Target sequence**<br>**(Probe A and Probe B)**	**Position**
TBP<br>**NM_001172085.1**	GCACGAAGTGCAATGGTCTTTAGGTCAAGTTTACAACCAAGATT<br>CACTGTCCTCAAGACCTAAGCGACAGCGTGACCTTGTTTCA<br>CGAAAGCCATGACCTCCGATCACTCTCCTCATGATTACCGCAGCA<br>AACCGCTTGGGATTATATTCGGCGTTTCGG	588-687
RPL13A<br>**NM_012423.2**	TCCTTGCTCCCAGCTTCCTATGTCCCAGGGCTGCCCATCCTCTTCT<br>TTTCTTGGTGTTGAGAAGATGCTC<br>CGAAAGCCATGACCTCCGATCACTCATTCTCCGAGTGCTTTCAAG<br>CAACTTCGGGAGGCAGTGACTAAGACCCTT	721-820
RPS17<br>**NM_001021.4**	CGGAGCTTTTTGCTGGGGATAATGGCGATCTCCTCGCACACGCGC<br>TTGTTCACAATTCTGCGGGTTAGCAGGAAGGTTAGGGAAC<br>CGAAAGCCATGACCTCCGATCACTCCTCTCTGAATTCGCTTCATC<br>AGATGCGTGACATAACCTGCTATCTTGTTG	207-306
RBFOX3<br>**NM_001082575.1**	ACCCAAAACCCTTGGAGCCCCGCTCGTTAAAAATGATCTCCACG<br>TCTAAACTGTTGAGATTATTGAGCTTCATCATGACCAGAAG<br>CGAAAGCCATGACCTCCGATCACTCTTCTCCCGGGCTCGGTCAG<br>CATCTGAGCTAGTTTCAAAAGTTACAA	577-676
MAP2<br>**NM_031845.2**	ACAGATTTGTAACAGTGTGTTGGAACCTCGGAATCCCAGCATAC<br>AGAGTACAAAGACGCCTATCTTCCAGTTTGATCGGGAAACT<br>CGAAAGCCATGACCTCCGATCACTCGCCTTTCTATGGTAACAGGC<br>TCTAGAATTATCAGAAGAAAGAAACCCCCC	5171-5270
SYN1<br>**NM_006950.3**	AGAGAATCCACCATTGGCATGGGCCACAAGGTTGAGATCAGAGA<br>ATTCGGCGAACCTAACTCCTCGCTACATTCCTATTGTTTTC<br>CGAAAGCCATGACCTCCGATCACTCTTCAGAGACCGCACGACCT<br>TCACCCCATTCCGAAGAACTTCCATATCCAC	566-665
DLG4<br>**NM_001365.3**	GTTTATACTGAGCGATGATCGTGACCGTCTGACCCGCATTCTTCA<br>GGGCACCAATTTGGTTTTACTCCCCTCGATTATGCGGAGT<br>CGAAAGCCATGACCTCCGATCACTCCCGAAGGTCGTGGATCTTG<br>GCCTCGAATCGGCTGTACTCTTCTG	2461-2560
CALB1<br>**NM_004929.2**	CCCCAGCACAGAGAATAAGAGCAAGATCCGTTCGGTACAGCTTC<br>CCCTTTCGGGTTATATCTATCAT<br>CGAAAGCCATGACCTCCGATCACTCTAGATACAGTGTATCACTAG<br>CAAGTGGTTGCGGCCACCAACTCTAGTTATTTACTTGACACCCT	911-1010
PVALB<br>**NM_002854.2**	CGAAGGAGTCGGTAGCGCTAAAGGCTCCCACCGCCTTCTTGATG<br>TCAACAGCCACTTTTTTTCCAAATTTTGCAAGAGCC<br>CGAAAGCCATGACCTCCGATCACTCATCCGCACTCTTTTTCTTCA<br>GGCCGACCATTTGGAAGAACTTTTTGTGGT	77-176
SLC17A7<br>**NM_020309.2**	GGCTAGGACCAGGAAGGAGATGGCCACGCCCTTGGCACCGTGT<br>GGACGGCAACTCAGAGATAACGCATAT<br>CGAAAGCCATGACCTCCGATCACTCTGGTTCACGTTGAACCCAG<br>AGATGGCGAAGCCGCTGAAGCCCAC	1331-1430
GAD1<br>**NM_000817.2**	TTGAAGGCACTCACAAGGCGACTCTTCTCTTCCAGGCTGTTGGT<br>CCTTTGCCTGGAGTTTATGTATTGCCAACGAGTTTGTCTTT<br>CGAAAGCCATGACCTCCGATCACTCCCCGGTCGCTGTTTTCACA<br>GGAAAGCAGGTTCTTGGAGGATTGCCTCTCC	576-675
GABRA1<br>**NM_000806.5**	GCTCAGAAGGAGGATCCAGGCCCAAAGACAGTCAGACAGACCA<br>GATAAGGTTGTTATTGTGGAGGATGTTACTACA<br>CGAAAGCCATGACCTCCGATCACTCTTAAGTTCATCTTGTAATGA<br>CGGCTGTCCATAGCTTCTTCCAGTCAGTGT	476-575
GFAP<br>**NM_002055.4**	CTCAATCTTCCTCTCCAGATCCAGACGGGCCAGGGCTGTTGAGAT<br>TATTGAGCTTCATCATGACCAGAAG<br>CGAAAGCCATGACCTCCGATCACTCCGTGGATCTTCCTCAAGAA<br>CCGGATCTCCTCCTCCAGCGA	590-689
ALDH1L1<br>**NM_012190.2**	TCCTTTTGCACGCCACCGGGAGTACTTGAATACCGGCACTCCATC<br>AAAGACGCCTATCTTCCAGTTTGATCGGGAAACT<br>CGAAAGCCATGACCTCCGATCACTCCCCCAAAGCCTGGTATTTTG<br>CCACCACATCAGGCAAAGCCTG	330-429

OLIG2<br>**NM_005806.2**	ATTGGATATGACCATCAGCGCTTCTGATACCGAACGCCGGCTTCC<br>AACTACGAACCTAACTCCTCGCTACATTCCTATTGTTTTC<br>CGAAAGCCATGACCTCCGATCACTCGGAGGAACGGCCACAGTTC<br>TAAGAGGGTGTGGATTGACCCAGATATTGAG	1691-1790
PROM1<br>**NM_006017.1**	CTTGATGGATGCACCAAGCACAGAGGGTCATTGAGAGATGACCG<br>CAGGCTCCAATTTGGTTTTACTCCCCTCGATTATGCGGAGT<br>CGAAAGCCATGACCTCCGATCACTCGGTTGCTATTCAGCTGGCTT<br>AGAGACAATCTGATGCTGTTGCAGGTTTCA	926-1025
NOTCH1<br>**NM_017617.3**	TATTTCAGATGCAAATTAATCCGCGTGCGGAAGGTGAGCCAGCTT<br>TGCCTCTTTCGGGTTATATCTATCATTTACTTGACACCCT<br>CGAAAGCCATGACCTCCGATCACTCCCATCTAAAACACATGGCA<br>ACATCTAACCCATATGCTTTCACTTGTTTCC	8212-8311
PAX6<br>**NM_000280.3**	CTCAAACTCTTTCTCCAGGGCCTCAATTTGCTCTTGGGTAAAGGA<br>TGTTCCAACAGCCACTTTTTTTCCAAATTTTGCAAGAGCC<br>CGAAAGCCATGACCTCCGATCACTCATTTTGGCTGCTAGTCTTTC<br>TCGGGCAAACACATCTGGATAATGGGTTCT	1174-1273
PDGFRB<br>**NM_002609.3**	CTGGTGCAGGCTCCTGAAGGCTCAGGAGAACAGAGGGATGCAC<br>CGTGTGGACGGCAACTCAGAGATAACGCATAT<br>CGAAAGCCATGACCTCCGATCACTCGTCCCAGAGTGGGTAACAG<br>CTGAGTAGAAGGACAGGCAGGA	266-365
CLDN5<br>**NM_001130861.1**	GGAAACTTCATTCCGTCTGTTAAGGGCAGGGCCGGGCTAGCCTG<br>GAGTTTATGTATTGCCAACGAGTTTGTCTTT<br>CGAAAGCCATGACCTCCGATCACTCCAGTCTGACACCCGCTCTG<br>CCTATGGAAACAGCGCCGCGCACAGAAAA	1924-2023
AIF1<br>**NM_032955.1**	CTCCCCGGAGCCACTGGACACCTCTCCAATTAATTTCTTTAGCTC<br>TAGGTCAGATAAGGTTGTTATTGTGGAGGATGTTACTACA<br>CGAAAGCCATGACCTCCGATCACTCGCAGATCTCTTGCCCAGCAT<br>CATCCTGAGAAAGTCAGGGTAGCTGAACGT	186-285

Supplementary Table 6: Table summarizing individual patient details for all the cases included in this study.

Cases (n = 49)	Age	Gender	Cortical region (n = 51)	Underlying Pathology
Tumour	60	Male	Parietal lobe	GBM
Tumour	52	Female	Parietal lobe	Anaplastic Astrocytoma III
Tumour	52	Male	Cerebellum	Metastatic tumour
Tumour	70	Male	Frontal lobe	GBM
Tumour	30	Female	Temporal lobe	Anaplastic Astrocytoma III
Tumour	57	Male	Temporal lobe	GBM
Tumour	57	Female	Parietal lobe	GBM
Tumour	55	Female	Temporal lobe	GBM
Tumour	62	Male	Temporal lobe	Anaplastic Astrocytoma III
Tumour	49	Female	Frontal lobe	Metastatic tumour
Tumour	75	Female	Temporal lobe	Diffuse Astrocytoma I-II
Tumour	58	Male	Cerebellum	Hemangioblastoma
Tumour	28	Female	Frontal lobe	Meningioma I-II
Tumour	23	Male	Temporal lobe	Metastatic tumour
Tumour	83	Male	Parietal lobe	GBM
Tumour	81	Male	Frontal lobe	GBM
Tumour	78	Female	Frontal lobe	GBM
Tumour	75	Male	Frontal lobe	GBM
Tumour	76	Male	Temporal lobe	GBM
Tumour	79	Male	Cerebellum	Hemangioblastoma
Tumour	46	Male	Cerebellum	Metastatic tumour
Tumour	24	Male	Frontal lobe	GBM
Tumour	20	Male	Cerebellum	Metastatic tumour
Tumour	64	Male	Frontal lobe	Metastatic tumour
Tumour	66	Male	Cerebellum	Meningioma
Tumour	66	Male	Occipital lobe	GBM
Tumour	58	Female	Cerebellum	Metastatic tumour
Tumour	52	Female	Frontal lobe	GBM
Epilepsy	52	Female	Temporal lobe Hippocampus	Intractable Epilepsy
Epilepsy	42	Female	Temporal lobe	Intractable Epilepsy
Epilepsy	30	Male	Temporal lobe	Intractable Epilepsy
Epilepsy	51	Female	Temporal lobe	Intractable Epilepsy
Epilepsy	40	Male	Temporal lobe	Intractable Epilepsy
Epilepsy	23	Male	Temporal lobe	Intractable Epilepsy
Epilepsy	35	Female	Temporal lobe	Intractable Epilepsy
Epilepsy	29	Female	Temporal lobe	Intractable Epilepsy

Epilepsy	36	Male	Temporal lobe	Intractable Epilepsy
Epilepsy	43	Male	Temporal lobe	Intractable Epilepsy
Paediatric	14	Female	Parietal lobe	Cortical dysplasia
Paediatric	7	Male	Frontal lobe	Cortical tuber
Paediatric	12	Female	Frontal lobe	Cortical dysplasia
Paediatric	5	Male	Parietal lobe	Cortical tuber
Paediatric	3	Male	Parietal lobe	Cortical dysplasia
Paediatric	9	Male	Parietal lobe	Cortical dysplasia
Paediatric	3	Female	Parietal lobe	Cortical tuber
Paediatric	10	Female	Frontal lobe	Intractable Epilepsy
Paediatric	3	Female	Frontal lobe	Cortical tuber
Paediatric	15	Male	Temporal lobe	Benign tumour
Paediatric	1	Male	Frontal lobe	Cortical dysplasia
Paediatric	4	Male	Parietal lobe	Cortical tuber

Supplementary Table 7: A summary of the number of cases used for each experimental paradigm.

**Experimental methods**	**Cases studied (total = 49)**	**Percentage**
IHC Analysis	21/49	43%
ICC Analysis	22/49	45%
RNA Analysis	14/49	29%
Electrophysiological Analysis	24/49	49%

Supplementary Table 8: The percentage of patch-clamp recorded cells from adult specimens showing active membrane properties (neuron or astrocyte-like traces) from each specimen type. Temporal lobe = Non-tumour epilepsy surgery, Peri-tumoural neocortex, Cerebellum = non-tumoural cerebellar cortex obtained in gaining access to brain stem complications.

**Region**	**AP Firing**		**Non-AP firing**
	Multi	Single	(Astrocyte-like)
**Temporal lobe (n=175)**	6 (4%)	90 (51%)	**79 (45%)**
**Peritumoral Neocortex (n=60)**	2 (3%)	30 (50%)	**28 (47%)**
**Cerebellum (n=143)**	36 (25%)	58 (41%)	**49 (34%)**
**Total cells (n=378)**	**44 (12%)**	**178 (47%)**	**156 (41%)**

Supplementary Table 9: The resting membrane potential (RMP) of recorded cells from each specimen type (same as above - adult cases only).

**Region/ Underlying Pathology**	**AP Firing (RMP ± SEM)**	**Non-AP firing (RMP ± SEM)**
**TL – Epilepsy (n=175)**	-66.9 ± 1.2 (n=96)	-75.5 ± 0.9 (n=79)
**Neocortex – Tumour (n=60)**	-62.1 ± 3.1 (n=32)	-74.3 ± 2.2 (n=28)
**Cerebellum – Tumour (n=143)**	-66.8 ± 1.4 (n=94)	-76.4 ± 1.1 (n=49)
**Total cells (n=378)**	-66.8 ± 0.9 (n=222)	-75.6 ± 0.7 (n=156)

Supplementary Table 10: Basal and active electrophysiological properties between paediatric (<16 years of age) and adult case (≥16 years of age)-derived neurons (exhibiting at least a single AP). For the average age and RMP, a two-tailed student t-test was conducted. For the percentage that exhibited multiple AP and synaptic activity, the difference in population proportions test was used.

**Age groups**	**Adult (n=222)**	**Paediatric (n=38)**
**Average age**	52.3 ± 3.1	7.5 ± 1.5 ($p < 0.001$)
**RMP ± SEM**	-66.6 ± 0.9	-61.6 ± 1.5 (ns)
**% Multiple AP**	44/222 = 19.8%	4/38 = 10.5% (ns)
**% Synaptic activity**	15/222 = 6.9%	4/38 = 10.5% (ns)
**Total neurons (n=260)**		
